# Huaier suppresses lung cancer by simultaneously and independently inhibiting the antioxidant pathway SLC7A11/GPX4 while enhancing ferritinophagy

**DOI:** 10.1038/s41420-025-02598-3

**Published:** 2025-07-07

**Authors:** Xingxing Shi, Kun Liu, Yuchang Tian, Xinyi Bi, Junkai Zhang, Fengyi Ma, Wensheng Wei, Tongbiao Zhao

**Affiliations:** 1 State Key Laboratory of Organ Regeneration and Reconstruction, Institute of Zoology, Chinese Academy of Sciences, Beijing, China; 2 Beijing Institute for Stem Cell and Regenerative Medicine, Beijing, China; 3 University of Chinese Academy of Sciences, Beijing, China; 4 Biomedical Pioneering Innovation Center, Peking-Tsinghua Center for Life Sciences, Peking University Genome Editing Research Center, State Key Laboratory of Gene Function and Modulation Research, School of Life Sciences, Peking University, Beijing, China

**Keywords:** Cell death, Drug development

## Abstract

Huaier (Trametes Robiniophila Murr), a traditional Chinese medicine, has emerged as a promising therapeutic agent against cancers in clinical settings, yet its underlying mechanisms remain elusive. In this study, we demonstrate that Huaier effectively suppresses lung cancer by inducing ferroptosis. Mechanistically, Huaier simultaneously and independently downregulates the antioxidant pathway SLC7A11/GPX4 and elevates intracellular iron levels through NCOA4-mediated ferritinophagy degradation of FTH1 in lung cancer cells. Both the iron chelator deferoxamine (DFO) and the ferroptosis inhibitor ferrostatin-1 (Fer-1) mitigate Huaier-induced cell death. In both urethane-induced lung tumorigenesis models and cell-derived xenograft (CDX) models, Huaier significantly inhibits tumor progression by inducing ferroptosis, which can be counteracted by SRS16-86. Our study uncovers a novel mechanism by which Huaier induces ferroptosis to suppress lung cancer, underscoring its potential as a therapeutic agent for lung cancer or as part of a combination therapy strategy.

## Introduction

Non-small cell lung cancer (NSCLC) accounts for ~85% of all lung cancer cases and remains the leading cause of cancer-related mortality worldwide, with a 5-year survival rate of less than 20% [1, 2]. Despite advances in targeted therapies, including EGFR inhibitors and immunotherapies such as PD-1/PD-L1 blockade, many patients eventually develop resistance, leading to disease progression and poor outcomes [3–6]. The need for novel therapeutic strategies that can overcome these resistance mechanisms is critical for improving long-term survival in NSCLC patients [7].

In recent years, ferroptosis, an iron-dependent form of regulated cell death distinct from apoptosis, necroptosis, and autophagy, has emerged as a promising therapeutic target in cancer treatment [8, 9]. Ferroptosis is characterized by the accumulation of lipid peroxides due to impaired glutathione metabolism and the inactivation of glutathione peroxidase 4 (GPX4), leading to oxidative damage and cell death [10]. GPX4 is a critical regulator of ferroptotic cell death [11, 12]; inhibiting GPX4 activity has been shown to sensitize cancer cells to ferroptosis and may help overcome resistance to conventional therapies [13, 14]. While recent studies suggest ferroptosis induction as a promising approach for treating cancer, its application is still in the early stages [15, 16].

Traditional Chinese Medicine (TCM) has a long history of use in cancer treatment, with several natural compounds showing promise as adjuvant therapies due to their ability to modulate various cellular processes, including cell proliferation, apoptosis, and immune responses [17]. Among these, Huaier (Trametes robiniophila Murr) is a well-known medicinal fungus in TCM, and has been utilized for over 1600 years in China18. The State Food and Drug Administration (SFDA) of the People’s Republic of China has approved it for clinical treatment of primary liver cancer (No. 023422) in China since 1997 [18]. Huaier has gained attention for its broad-spectrum anti-cancer effects in cancers such as hepatocellular carcinoma, breast cancer, and colorectal cancer [19–24]. Currently, Huaier’s anti-cancer activity has been attributed to its ability to regulate multiple signaling pathways, including PI3K/AKT, MAPK, and NF-κB, as well as its capacity to modulate immune responses and inhibit metastasis [25, 26].

Based on the positive effects demonstrated by Huaier in clinical practice against the aforementioned series of cancers, this study explores the antitumor effects of Huaier on non-small cell lung cancer (NSCLC) and its mechanisms targeting cancer cell death. Given Huaier’s direct application in clinical settings for treating certain cancers, we initially demonstrated through a primary lung cancer model that Huaier treatment significantly inhibited the growth of lung cancer tissues and extended the survival of tumor-bearing mice, indicating a notable therapeutic effect on NSCLC. Furthermore, we showed that Huaier induces ferroptosis in lung cancer cells by concurrently downregulating the SLC7A11/GPX4 signaling pathway, which reduces the antioxidant capacity of cancer cells, and upregulating the ferritinophagy signaling pathway, thereby increasing the oxidative stress within cancer cells. These findings highlight the potential for clinical applications of Huaier in the treatment of lung cancer.

## Results

### Huaier inhibits NSCLC progression

**Fig. 1:**
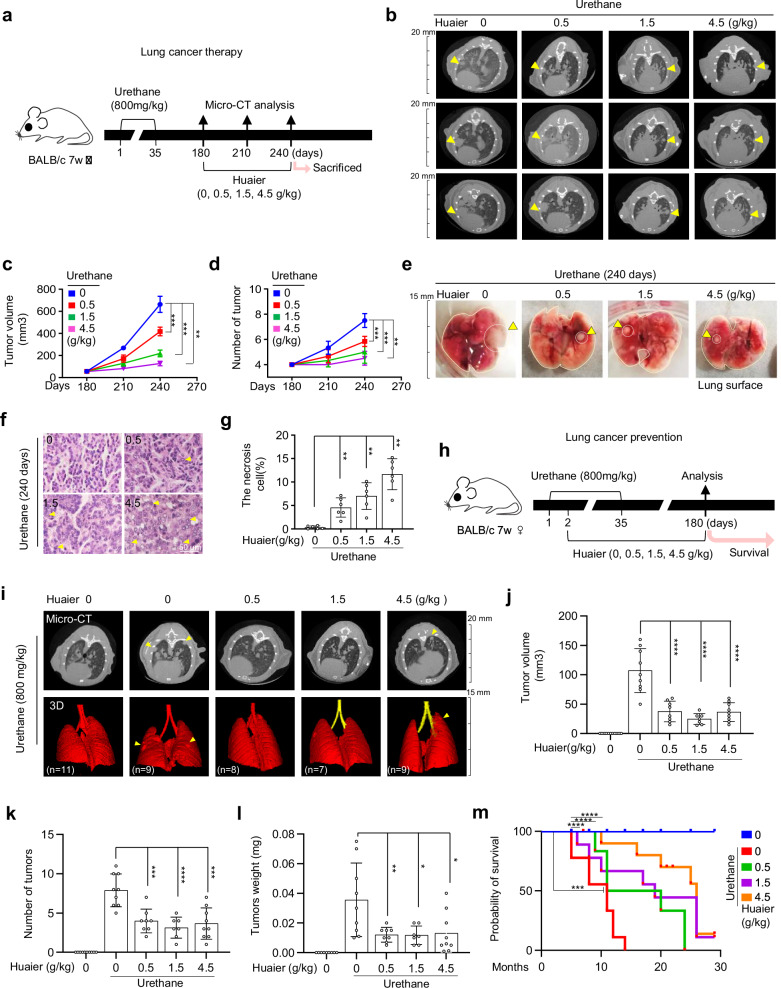
Huaier inhibits the progression of lung cancer. **a** Schematic timeline of the lung cancer therapy strategy, showing urethane, Huaier, or vehicle (0.9% NaCl) treatment and Micro-CT scans at 180, 210, and 240 days. **b** Micro-CT images at 180, 210, and 240 days, with yellow triangles indicating primary lung tumors. *n* = 6. **c**, **d** Tumor volume and count from Micro-CT scans of mice at 180, 210, and 240 days. Data are shown as mean ± SD, *n* = 6, **p < 0.01; ***p < 0.001. **e**, **f**, **g** At 240 days post-treatment, lung tissues were isolated and analyzed: **e** Representative images lung surface, with yellow triangles marking primary lung tumors. **f** H&E staining and necrosis in lung tumor sections from urethane-treated mice, with or without Huaier treatment; yellow triangles highlight necrotic regions. *n* = 6. Scale bar: 50 µm. **g** Quantification of necrotic regions. Data are shown as mean ± SD, *n* = 6, **p < 0.01. **h** Schematic representation of Huaier administration protocols in the urethane-induced lung cancer mouse model. **i** Micro-CT images and 3D reconstructions of lungs from Huaier-treated (0.5, 1.5, 4.5 g/kg) urethane-induced mice at 180 days, with yellow triangles indicating primary lung tumors. *n* ≥ 7. **j** Tumor volume in mice scanned by Micro-CT. Data are shown as mean ± SD, *n* ≥ 7 per group, ****p < 0.0001. **k**, **l** Tumor counts and weight in urethane-treated mice. Data were shown as mean ± SD, *n* ≥ 7 per group, *p < 0.05; **p < 0.01, ***p < 0.001, ****p < 0.0001. **m** Lifespan of mice treated with urethane, with or without Huaier. Data were shown as mean ± SD, *n* = 10 mice per group, ***p < 0.001, ****p < 0.0001.

To assess the antitumor efficacy of Huaier on lung cancer in vivo, we initially developed a primary lung tumor model utilizing urethane administration as previously documented [27–29]. Two treatment strategies were implemented: lung cancer therapy and prevention (Fig. 1a, h). In the therapeutic approach, Balb/c mice received urethane at dose of 800 mg/kg twice weekly for 5 weeks via intraperitoneal injection in 0.9% NaCl. These mice with primary lung tumors (180 days after Urethane administration) were then treated with Huaier. Tumor harboring mice were grouped and intragastric administered with Huaier at dosages of 0.5, 1.5, and 4.5 g/kg daily for 2 months. Micro-CT scans revealed that Huaier treatment for 1 and 2 months (at 210 and 240 days) significantly reduced tumor growth, volume, and number in a dose-dependent manner (Fig. 1b–d). Upon sacrifice at 240 days, analysis of lung tumor tissue confirmed that Huaier-treated mice exhibited smaller tumors compared to the vehicle group (Figs. 1e and S1a). Histological examination using H&E staining indicated enhanced tumor cell death in Huaier-treated mice, characterized by large necrotic regions (Fig. 1f, g).

In the prevention strategy, mice were administered with Huaier at dosages of 0.5, 1.5, and 4.5 g/kg or vehicle (0.9% NaCl) daily via gavage since the second day of urethane injections (Fig. 1h). After 6 months, Micro-CT imaging on these mice revealed a significant reduction in the number of visible lung tumors in Huaier-treated mice compared to vehicle-treated controls (Fig. 1i). Further analysis showed a marked decrease in tumor volume, number, and weight in the Huaier-treated groups (Fig. 1j–l). Consistent with these observations, Huaier treatment improved mouse survival, with median survival durations of 11 months for the vehicle group and 26 months for the Huaier (4.5 g/kg)-treated group, extending median survival by 15 months (Fig. 1m). Meanwhile, in our long-term toxicity experiments (as shown in Fig. S1b–e), both the body weight of the mice (Fig. S1c) and the histological examination of liver and kidney tissues using H&E staining (Fig. S1d, e) demonstrated that Huaier administration did not result in any significant toxic effect.

Next, the direct inhibitory effect of Huaier on cancer cells was evaluated in vitro using three NSCLC cell lines: A549, H1975, and H358. Exposure to Huaier at varying concentrations for 48 h resulted in dose-dependent NSCLC cell death (Fig. S2a, b). Collectively, these findings indicate that Huaier effectively inhibits lung cancer progression, at least partially through the direct suppression of lung cancer cell survival.

### Huaier induces ferroptosis in NSCLC cells

**Fig. 2:**
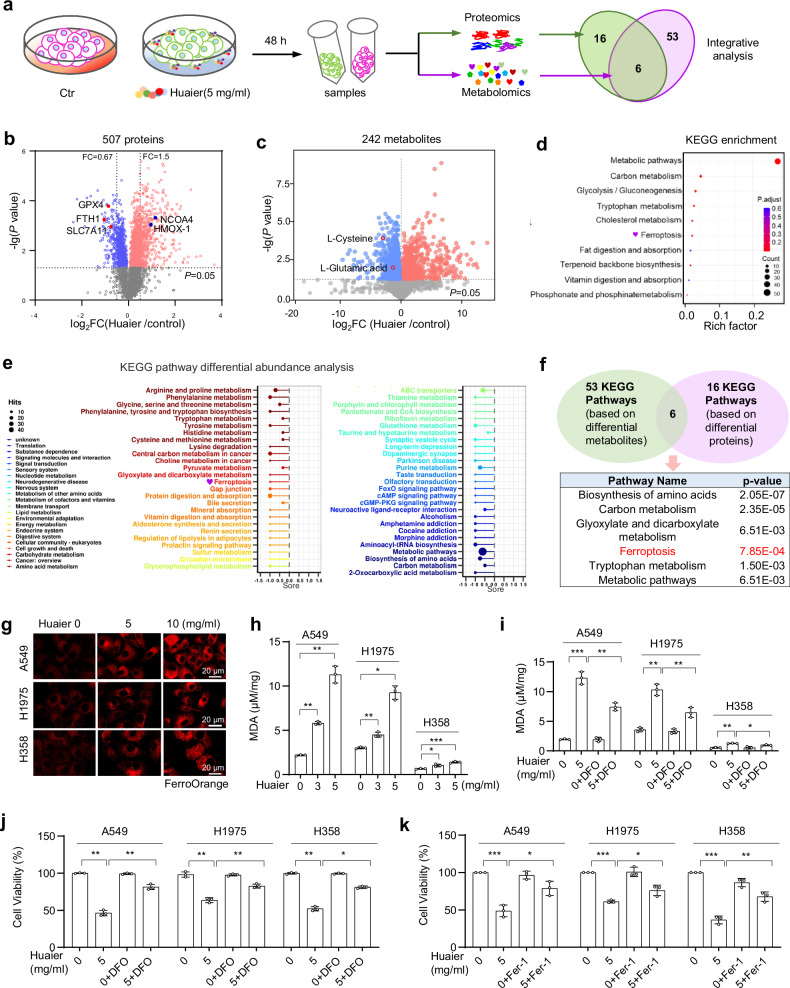
Huaier induces ferroptosis in lung cancer cells. **a** Schematic diagram of the omics experiments. A549 cells were treated with Huaier (5 mg/ml) for 48 h, followed by proteome and metabolome analysis. Volcano plots display the adjusted *p*-value and the log2 Fold Change (FC) of proteins (**b**) and metabolites (**c**) from Huaier-treated A549 cells compared to control omics data. **d** Enrichment analysis of differentially expressed proteins was conducted using the Kyoto Encyclopedia of Genes and Genomes (KEGG) pathway. **e** KEGG pathway differential abundance analysis of metabolome. **f** KEGG pathway enrichment integrated analyses of the proteome and metabolome are illustrated in a Venn diagram. **g** Confocal imaging FerroOrange (red) in Huaier-treated NSCLC cells. A549, H1975 and H358 cells were treated with Huaier (5 or 10 mg/ml) for 48 h, and samples were prepared for confocal imaging. Scale bar: 20 μm. **h** Intracellular MDA levels in Huaier-treated NSCLC cells. A549, H1975 and H358 cells were treated with Huaier (5 or 10 mg/ml) for 48 h, followed by MDA content analysis. Data are shown as mean ± SD, *n* = 3, *p < 0.05, **p < 0.01, ***p < 0.001. **i** Intracellular MDA levels and (**j**) cell viability were assessed in A549, H1975, and H358 cells treated with Huaier (5 mg/ml) in the absence or presence of Deferoxamine (DFO) (1 μM) for 48 h. Data are shown as mean ± SD, *n* = 3, *p < 0.05, **p < 0.01. **k** A549, H1975, and H358 cells were treated with Huaier (5 mg/ml) in the absence or presence of Ferrostatin-1 (Fer-1) (2 μM) for 48 h, and cell viability was measured. Data are shown as mean ± SD, *n* = 3, *p < 0.05, **p < 0.01, ***p < 0.001.

To elucidate the molecular mechanisms underlying Huaier-induced NSCLC cell death, we employed tandem mass tag (TMT)-based quantitative proteomics and non-targeted metabolomics analyses (Fig. 2a). This integrated approach identified a total of 507 proteins and 242 metabolites exhibiting significant differential expression following Huaier treatment (Fig. 2b, c). KEGG pathway analysis revealed that these proteins and metabolites were predominantly enriched in various metabolic processes, including a notable emphasis on ferroptosis (Fig. 2d, e).

By integrating the proteomic and metabolomic datasets, we identified ferroptosis as a prominent candidate pathway mediating Huaier-induced cell death (Fig. 2f and Tables S1–S3). The ferroptosis pathway was significantly enriched in both omics analyses, suggesting its critical role in the observed cellular responses. This integrative analysis highlights ferroptosis as a potential mechanism through which Huaier exerts its antitumor effects on NSCLC cells.

To experimentally validate the role of ferroptosis in Huaier-induced cancer cell suppression, we assessed several ferroptosis indicators in Huaier-treated NSCLC cells, including A549, H1299, and H358. Huaier treatment (5–10 mg/ml for 48 h) significantly increased the accumulation of Fe^2+^, malondialdehyde (MDA), and lipid peroxidation, and enhanced LDH and ROS production (Figs. 2g, h, and S2c–g). Furthermore, the application of ferroptosis inhibitors, such as DFO and Fer-1, successfully mitigated Huaier-induced MDA accumulation and cell death, providing compelling evidence that Huaier induces ferroptosis in NSCLC cells (Figs. 2i–k, and S2h).

### Huaier downregulates SLC7A11/GPX4 axis in lung cancer cells

**Fig. 3:**
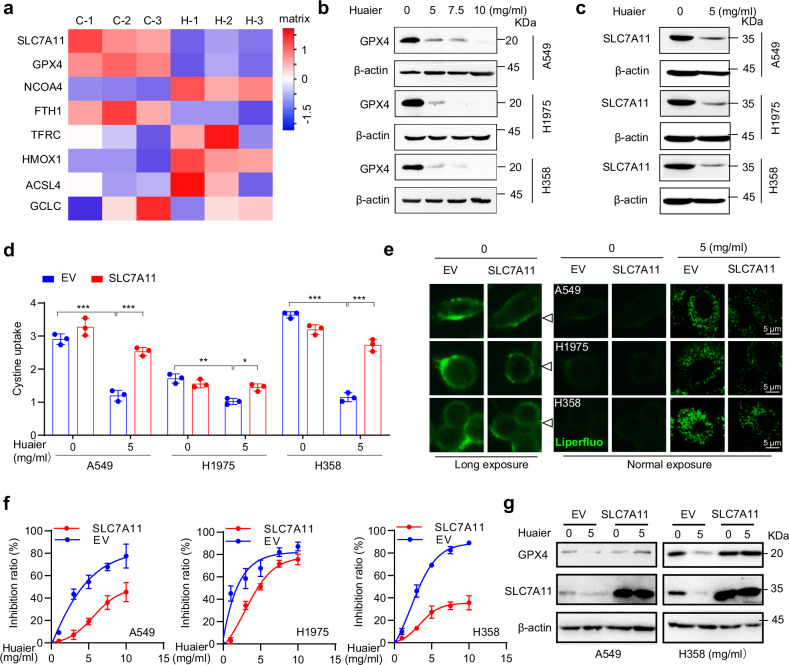
Huaier induces downregulation of the SLC7A11/GPX4 axis. **a** Heatmap illustrating the differentially expressed proteins involved in the ferroptosis pathway in Huaier-treated vs control A549 cells. Samples are labeled as C 1–3 (Control 1–3), H 1-3 (Huaier 1–3). **b** Western blot analysis of GPX4 expression in A549, H1975, and H358 cells following treatment with Huaier at concentrations of 0, 5, 7.5, and 10 mg/ml for 48 h. **c** Western blot analysis of SLC7A11 expression in A549, H1975, and H358 cells treated with Huaier at 5 mg/ml for 48 h. A549, H1975, and H358 cells were stably transfected with SLC7A11 plasmid and subsequently treated with Huaier at 5 mg/ml for 48 h. Cystine uptake (**d**) and Lipid peroxidation (**e**) were assessed. Scale bar: 5 μm. Data are shown as mean ± SD, *n* = 3, *p < 0.05, **p < 0.01, ***p < 0.001. **f** A549, H1975, and H358 cells, stably transfected with SLC7A11 plasmid, were treated with varying Huaier concentrations of Huaier (0, 1, 3, 5, 7.5, 10 mg/ml) for 48 h, and the inhibition ratio was determined using a CCK-8 assay. **g** In A549 and H358 cells, stably transfected with the SLC7A11 plasmid and treated with Huaier at 5 mg/ml for 48 h, Western blot analysis was performed to evaluate SLC7A11 and GPX4 protein levels.

To further investigate the regulatory mechanisms of Huaier on ferroptosis, we conducted a comprehensive proteomic analysis focusing on ferroptosis-related proteins. Our findings identified SLC7A11, GPX4, NCOA4, and FTH1 as crucial mediators potentially involved in Huaier-induced ferroptosis in NSCLC cells (Fig. 3a and Table S4). Western blot and immunofluorescence staining revealed a marked reduction in the levels of SLC7A11 and GPX4 in Huaier-treated NSCLC cells (Figs. 3b, c, and S3a, b). Further analysis demonstrated that Huaier treatment led to a reduction in cystine uptake and an increase in intracellular glutamate levels, indicating a suppression of the system Xc^−^ transporter function (Figs. 3d, and S3c, d). This transporter is critical for importing cystine, which is essential for glutathione biosynthesis and antioxidant defense [30]. Knockdown of GPX4 further enhanced Huaier-induced cell death in NSCLC cells, and this effect was amplified by the GPX4 inhibitor RSL3 (Fig. S3e–i). These results support the hypothesis that Huaier induces ferroptosis in NSCLC cells by inhibiting the activity of SLC7A11 and GPX4, thereby disrupting the cellular antioxidant defense mechanisms.

To validate this hypothesis, we developed NSCLC cell lines (A549, H1975, and H358) with stable overexpression of SLC7A11 or GPX4 (Fig. S3j, k). Overexpression of these proteins significantly counteracted the effects of Huaier, restoring cystine uptake, reducing lipid peroxidation accumulation, and maintaining GPX4 expression levels in NSCLC cells (Figs. 3d–g, and S3l–n). These findings underscore the critical role of the SLC7A11/GPX4 signaling pathway in Huaier-induced ferroptosis. Collectively, our data suggest that Huaier exerts its antitumor effects in NSCLC cells by targeting and modulating this specific ferroptosis pathway, offering insights into potential therapeutic strategies for lung cancer treatment.

### Huaier induces ferritinophagy in lung cancer cells by promoting NCOA4-mediated degradation of FTH1

Ferroptosis is recognized as an autophagy-dependent form of cell death, with a specific autophagy pathway known as ferritinophagy playing a critical role in this process [31–33]. We observed significant alterations in the expression of NCOA4 and FTH1 in NSCLC cells treated with Huaier, suggesting their involvement in ferritinophagy (Fig. 3a and Table S4). Ferritinophagy is mediated by NCOA4, which specifically recognizes FTH1 and facilitates its transport to autophagosomes for lysosomal degradation, leading to the release of iron [12, 32, 34, 35]. These findings led us to hypothesize that Huaier-induced ferritinophagy could act through NCOA4/FTH1 in NSCLC cells.

**Fig. 4:**
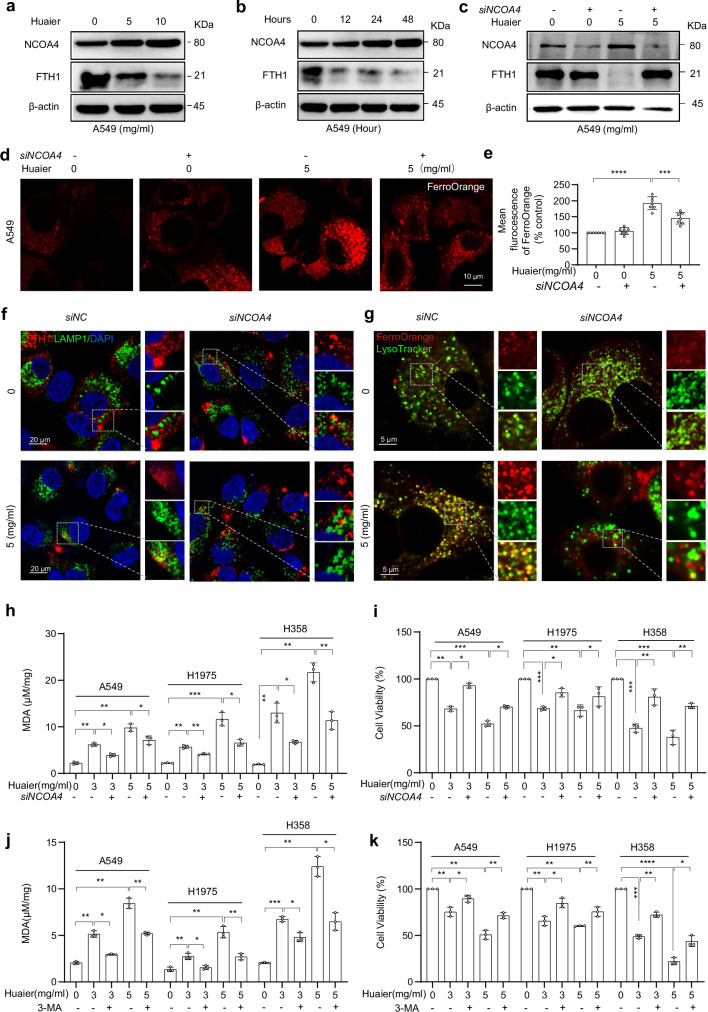
Huaier induces ferritinophagy in lung cancer cells by promoting the degradation of FTH1 via NCOA4. **a** Western blot analysis of NCOA4 and FTH1 expression in A549 cells treated with Huaier at concentrations of 0, 5, 10 mg/ml for 48 h. **b** Western blot analysis of NCOA4 and FTH1 expression in A549 cells treated with 5 mg/ml Huaier for 0, 12, 24, and 48 h. **c**–**g** A549 cells were transfected with 100 nM of specific NCOA4 siRNA (*siNCOA4*), and subsequently treated with 5 mg/ml Huaier for 48 h. **c** Western blot analysis of NCOA4 and FTH1 protein levels. **d** Confocal imaging of FerroOrange (red); Scale bar: 10 μm. **e** Quantification of FerroOrange fluorescence intensity Data are shown as mean ± SD, *n* = 7, ***p < 0.001, ****p < 0.0001. **f** Confocal imaging of FIH1 (red) and LAMP1 (green) in A549 cells treated with 5 mg/ml Huaier for 48 h. LAMP1 (green) stains lysosomes, with colocalized foci indicated by white boxes and shown in an enlarged image; scale bar: 20 μm. **g** Confocal imaging of FerroOrange (red) and LysoTracker Green (green) in A549 cells treated with 5 mg/ml Huaier for 48 h. Colocalized foci are indicated by white boxes and shown in an enlarged image; scale bar: 5 μm. **h**, **i** A549 cells were transfected with 100 nM *siNCOA4* and treated with 3 or 5 mg/ml Huaier for 48 h. **h** Intracellular MDA content and **i** Cell viability were assessed. Data are shown as mean ± SD, *n* = 3, *p < 0.05, **p < 0.01, ***p < 0.001. **j** Intracellular MDA content and **k** cell viability were measured in A549, H1975, and H358 cells treated with 3 or 5 mg/ml Huaier, with or without 3-MA (5 mM) for 48 h. Data are shown as mean ± SD, *n* = 3, *p < 0.05, **p < 0.01, ***p < 0.001, ****p < 0.0001.

Upon further investigation, we found that Huaier treatment resulted in a significant increase in NCOA4 protein levels and a decrease in FTH1 protein levels in the three distinct lung cancer cell lines consistently (Figs. 4a, b, and S4a–d), but did not alter the transcription level of these genes in these cell lines (Fig. S4e). Immunofluorescence analyses revealed enhanced colocalization of FTH1 with lysosomes and autophagosomes, as well as increased colocalization of NCOA4 with autophagosomes following Huaier treatment (Fig. S4f–h). Additionally, there was a notable accumulation of Fe^2+^ in lysosomes, as indicated by the colocalization of FerroOrange and LysoTracker Green (Fig. S4i, j). These observations suggest that the degradation of NCOA4 and FTH1 via the autophagy-lysosomal system leads to the release of ferrous iron, a key event in the induction of ferroptosis. The use of autophagy inhibitors, such as 3-methyladenine (3-MA) and Bfilomycin A1 (BafA1), significantly inhibited Huaier-induced FTH1 degradation, further supporting the role of ferritinophagy in this process (Fig. S4k, l).

To further confirm the role of NCOA4-mediated degradation of FTH1 in Huaier-induced ferroptosis, we employed NCOA4 small interference RNA (siRNA) assays (Fig. S5a). Knockdown of NCOA4 effectively reversed the reduction of FTH1, the accumulation of Fe^2+^, and the colocalization of Fe^2+^ or FTH1 with lysosomes in NSCLC cells treated with Huaier (Figs. 4c–g and S5b). Moreover, the lipid peroxidation, malondialdehyde (MDA) accumulation, and the inhibitory effects of Huaier on NSCLC cells were also mitigated by NCOA4 knockdown or autophagy inhibition (Figs. 4h–k and S5c, d). These findings underscore the importance of NCOA4-mediated, autophagy-dependent degradation of FTH1 in the induction of ferroptosis by Huaier in NSCLC cells.

### Huaier independently downregulates the SLC7A11/GPX4 axis and upregulates ferritinophagy

Recent studies have demonstrated that autophagy inhibition can lead to the inactivation of SLC7A11 through an mTOR-mediated mechanism [36], while chaperone-mediated autophagy contributes to GPX4 degradation, ultimately promoting ferroptosis [37]. In light of these findings, we explored the potential connection between the SLC7A11/GPX4 signaling pathway and ferritinophagy. To this end, we treated cells overexpressing SLC7A11 with Huaier at a concentration of 5 mg/ml and subsequently assessed the expression levels of NCOA4, FTH1, and LC3B-II. Notably, no statistically significant alterations in these markers were observed relative to empty vector (EV) controls, suggesting functional independence between these pathways (Fig. S5e).

To further dissect this relationship, we investigated the impact of NCOA4 knockdown and Bafilomycin A1 (BafA1) treatment on the expression of SLC7A11 and GPX4 in response to Huaier treatment. Strikingly, neither BafA1 blockade of autophagic flux nor NCOA4 knockdown affected the expression level of SLC7A11 and GPX4 after 48 h of exposure to Huaier at 5 mg/ml (Fig. S5f–h). These findings suggest that the SLC7A11/GPX4 signaling pathway does not intersect with ferritinophagy in the context of Huaier-induced ferroptosis.

These comprehensive analyses together indicate that ferritinophagy operates independently of the SLC7A11/GPX4 signaling pathway during Huaier-induced ferroptosis. This independence underscores the complexity of the molecular mechanisms involved in ferroptosis and highlights the distinct pathways through which Huaier exerts its antitumor effects.

### Huaier suppresses lung tumor progression by inducing ferroptosis

**Fig. 5:**
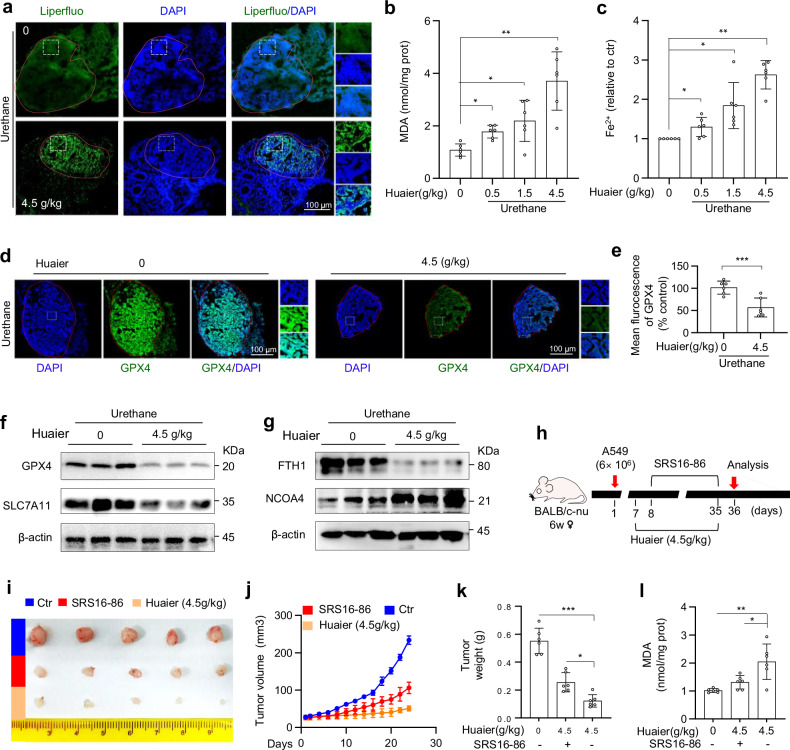
Huaier suppresses lung cancer progression by inducing ferroptosis. **a–g** Lung tissues from the mice treated as Fig. 1a were isolated at day 240 and analyzed. **a** Confocal image of Liperfluo fluorescence intensity. Scale bar: 100 μm. **b**, **c** The contents of MDA and Fe^2+^ in lung tumor nodules were measured. *n* = 6 mice per group. Data are shown as mean ± SD, *n* = 6, *p < 0.05, **p < 0.01. **d** Confocal microscopy analysis of GPX4 expression in tumor tissues. The GPX4 expressions are indicated by white boxes and shown in an enlarged image. Scale bar: 100 μm. **e** Quantification of the fluorescence intensity of GPX4. Data are shown as mean ± SD, *n* = 6, ***p < 0.001. **f**, **g** The expression levels of GPX4, SLC7A11, FTH1 and NOCA4 in tumor tissues were determined by western blot. *n* = 3 mice per group. **h** A schematic diagram illustrates the establishment of A549 xenograft in immune-deficient BALB/c nude mice. These mice were treated daily with Huaier at 4.5 g/kg by gavage for 24 consecutive days, while SRS16-86 (2 mg/kg) was administered twice per week. **i** Images of tumor samples from each group are shown, and **j** the tumor volume was calculated at indicated time points post-treatment. Tumor weight (**k**) and MDA contents (**l**) were also measured. *n* = 6 mice per group. Data are shown as mean ± SD, *n* = 6, *p < 0.05, **p < 0.01, ***p < 0.001.

To investigate whether Huaier suppresses lung tumor progression through ferroptosis in vivo, we employed the primary lung tumor models. Following two months of Huaier treatment on these tumor-bearing mice, we observed significant alterations in ferroptosis markers. Specifically, Huaier-treated lung tumors exhibited substantial accumulation of lipid peroxidation, malondialdehyde (MDA), and Fe^2+^, indicating the induction of ferroptosis in vivo (Fig. 5a–c). Notably, these tumors also demonstrated inhibition of the SLC7A11/GPX4 pathway and activation of the NCOA4/FTH1 pathway, suggesting a dual mechanism of action (Figs. 5d–g and S5i).

In CDX models, we further explored the effects of Huaier when combination with SRS16-86, a third-generation small molecule ferroptosis-specific inhibitor that targets lipid reactive oxygen species (ROS) (Fig. 5h). Our data revealed that Huaier alone effectively suppressed lung tumor growth and induced MDA accumulation (Fig. 5i–l). However, the addition of SRS16-86 attenuated the anti-tumor efficacy of Huaier (Fig. 5i–l), underscoring the role of ferroptosis in Huaier’s mechanism of action in vivo.

## Discussion

**Fig. 6:**
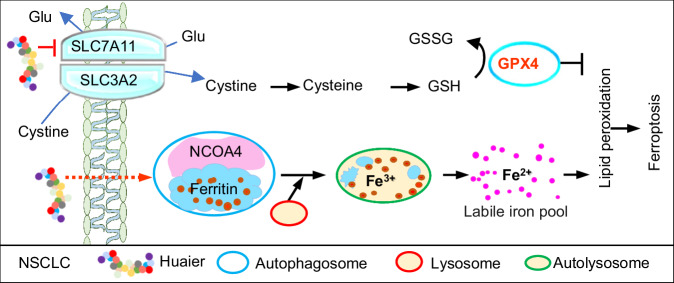
Mechanism of Huaier action in lung cancer suppression. A schematic diagram illustrating the central role of Huaier in inducing ferroptosis in NSCLC. Huaier supresses the expression of SLC7A11 and GPX4, thereby inactivating the SLC7A11/GPX4 signaling axis and reducing cancers cells‘ resistance to ferroptosis. Additionally, Huaier facilitates the degradation of FTH1 through NCOA4, leading to increased iron accumulation. The combined effects of Huaier-mediated SLC7A11/GPX4 inhibition and enhanced ferritinophagy synergistically drive NSCLC cells towards ferroptosis.

In this study, we demonstrated that Huaier effectively induces ferroptosis in non-small cell lung cancer cells to suppress cancer progression. Mechanistically, Huaier triggers ferroptosis in lung cancer cells by simultaneously disrupting the redox homeostasis and enhancing ferritinophagy. On the one hand, Huaier significantly reduces the antioxidant capacity of lung cancer cells by downregulating the SLC7A11/GPX4 pathway, thereby increasing their sensitivity to ferroptosis. On the other hand, Huaier enhances NCOA4-mediated degradation of FTH1 ferritin, increasing the intracellular labile iron pool. The ferrous ion, in turn, catalyzes the production of reactive oxygen species (ROS) through the Fenton reaction, ultimately leading to the oxidation of membrane lipid molecules and inducing ferroptosis in tumor cells (Fig. 6). Interestingly, Huaier can simultaneously and independently target different intracellular molecular pathways that converge on the induction of ferroptosis in target cells, highlighting Huaier’s ability, as a TCM, to act on multiple targets within the same tumor cell.

Notably, the antitumor efficacy of Huaier has been validated in vivo using both urethane-induced primary lung tumor models and cell-derived xenograft (CDX) models. In these models, Huaier treatment significantly inhibited tumor growth, indicating its potential as a therapeutic agent for NSCLC. These in vivo findings are particularly relevant as they recapitulate the complexity of tumor biology in a physiological setting, offering a more comprehensive understanding of Huaier’s anticancer mechanisms and supporting its potential clinical application. In these models, Huaier’s ability to inhibit tumor progression by inducing ferroptosis suggests that it could not only serve as an adjunct to traditional therapies—such as chemotherapy, targeted therapy, immune checkpoint inhibitor therapy, and surgical resection—to enhance treatment efficacy, but also holds promise as a standalone therapy for cancers. This warrants further validation through large animal trials and clinical studies.

Ferroptosis is a promising therapeutic target in cancers characterized by high oxidative stress and metabolic vulnerability. Like many other solid tumors, non-small cell lung cancer often develops resistance to conventional therapies such as chemotherapy, targeted therapy, and immune checkpoint inhibitors. Recent preliminary in vitro studies have shown that NSCLC responds to certain compounds, such as artemisinin, by undergoing ferroptosis [38]. This suggests that inducing ferroptosis could become a new treatment strategy for refractory lung cancers resistant to classical apoptosis-inducing drugs. Huaier granules, approved by the China National Medical Products Administration, can be used alone or in combination with other drugs for the treatment of various cancers. A recent multicenter, randomized, controlled Phase IV clinical trial demonstrated that Huaier granules, as adjuvant therapy after curative liver resection for hepatocellular carcinoma (HCC), significantly prolonged recurrence-free survival (RFS) and reduced extrahepatic recurrence. Our findings on Huaier, along with its good performance in clinical treatment of various cancers, support the clinical application of Huaier in treating NSCLC.

In summary, our study demonstrates that Huaier effectively induces ferroptosis in NSCLC by simultaneously modulating the SLC7A11/GPX4 axis and ferritinophagy, thereby exerting its anti-tumor effects both in vitro and in vivo. By targeting multiple molecular pathways that inducing ferroptosis, Huaier emerges as a promising therapeutic candidate, particularly for patients with resistant NSCLC.

### Limitations

Despite the encouraging results, our study has several limitations that warrant further investigation. First, although we demonstrated that Huaier induces ferroptosis in tumor cells by downregulating the SLC7A11/GPX4 pathway and upregulating ferritinophagy in both in vitro and in vivo models, the exact mechanisms by which Huaier causes the downregulation of SLC7A11 and the upregulation of NCOA4 require further elucidation. Second, the pharmacokinetics and optimal dosing strategy of Huaier in human patients remain to be determined. Preclinical pharmacokinetic studies are needed to determine the bioavailability and therapeutic window of Huaier to ensure its safety and efficacy in clinical settings. Thirdly, the potential benefits of combining Huaier with other cancer therapies should be explored, as targeting multiple cell death pathways simultaneously may enhance therapeutic effects and overcome resistance. Fourthly, the metabolic processes and pharmacokinetic characteristics of Huaier in animals need to be determined. Finally, clinical translation need to be further integrated with human sample research. Our team is committed to focusing on these key directions in future studies to enhance the translational relevance of this work.

## Materials and methods

### Cell line and culture conditions

Human non-small cell lung carcinoma (NSCLC) cell lines, including NCI-H1975, A549, and NCI-H358, as well as human embryonic kidney 293T cells, were obtained from the American Type Culture Collection (ATCC) in Rockville, MD, USA. These cells were cultured in either Dulbecco’s Modified Eagle’s Medium (DMEM) or Roswell Park Memorial Institute (RPMI)-1640 (Gibco, Waltham, MA, USA), both supplemented with 10% fetal bovine serum (FBS) (VisTech, Sydney, Australia), 100 μg/ml streptomycin, and 100 μ/ml penicillin. These cultures were maintained in an incubator at 37 °C in 5% CO_2_.

### Preparation of Huaier reagents

The purification of Huaier (Gaitianli, Jiangsu, China) involves the following steps: (1) Take the Huaier substrate and decoct it with water three times. For the first decoction, add 7 times the amount of water, boil for 1.5 h, filter, and reserve the filtrate. (2) For the second and third decoctions, add 5 times the amount of water each time, boil for 1 h, and filter. (3) Combine all three filtrates, concentrate under reduced pressure to a relative density of 1.35–1.40 at 55 °C, and obtain the final product, Huaier.

Huaier was dissolved in phosphate-buffered saline (PBS) and filtered through a 0.22 μm filter to create a 100 mg/ml stock solution. This stock solution was subsequently diluted to the desired working concentration prior to use. In the Huaier treatment group, A549 cells were incubated with Huaier, while the control group received an equivalent volume of complete DMEM or RPMI-1640 with elevated glucose levels. Details of the reagents used in this study are provided in Table S5.

### Immunofluorescence staining

Cells were fixed with 4% (w/v) paraformaldehyde for 15 min and permeabilized with 0.5% Triton X-100 for 20 min at room temperature. They were then incubated with primary antibodies overnight at 4 °C, followed by secondary antibodies at room temperature for 60 min. Nuclei were counterstained with DAPI. For tissue immunofluorescence staining, tumor tissues were fixed in 4% paraformaldehyde (PFA) for 1 h at 4 °C. After washing three times in PBS, the tissues were dehydrated in 30% sucrose overnight and embedded in OCT compound (Sakura). Cryosections (~10 µm thick) were collected on negatively charged slides and stored at −20 °C until use. The dried sections were washed twice with PBS, blocked with 5% normal donkey serum in PBST (0.2% Triton X-100 in PBS), and stained with primary antibodies at 4 °C overnight. After washing with PBS three times, sections were incubated with Alexa-conjugated secondary antibodies (Invitrogen) for 60 min at room temperature. Sections were then washed with PBS three times and mounted with a mounting medium containing DAPI (Vector Labs). Digital images were captured and analyzed. The antibodies used in this study are listed in Table S6.

### Western blotting

Cells were lysed in a lysis buffer containing 50 mM Tris-HCl (pH 7.4), 150 mM NaCl, 1% NP-40, 0.5% sodium deoxycholate, 0.1% SDS, and a protease inhibitor cocktail. Equivalent amounts of protein were subjected to SDS-PAGE and transferred onto PVDF membranes (Millipore). Membranes were blocked with 5% non-fat milk for 1 h at room temperature, then incubated with the specified primary antibodies overnight at 4 °C. This was followed by incubation with the appropriate HRP-conjugated secondary antibodies for 1 h at room temperature. After washing with TBST three times, the membranes were visualized with chemiluminescent kits (Millipore). The antibodies used in this study are listed in Table S6.

### Quantitative real-time PCR

Total RNA was extracted from samples using the RNeasy Total RNA Isolation Kit (Qiagen, 74104). The extracted RNA was then reverse transcribed into cDNA using a SuperScriptTM III First-Strand Synthesis System (Invitrogen Thermo Fisher Scientific, 18080051) according to the manufacturer’s instructions. The primer sequences specific to each gene are listed in Table S7.

### Cell viability assay

The viability of A549, H1975, and H358 cells following Huaier treatments was determined using the CCK8 assay kits (Dojindo, CK04). Approximately 5 × 10^3^ cells per well were seeded into 96-well plates and treated with various concentrations of Huaier, with or without inhibitor or inducer, for 48 h. After treatment, 100 μ of medium containing 10% CCK-8 was added to each well and incubated at 37 °C for 4 h. Absorbance was measured at a wavelength of 450 nm.

### SiRNA-mediated gene knockdown

Cells were plated in 96-well microplates at a density of 5000 cells per well and transfected with gene-targeting siRNA (synthesized by Genepharma) or scrambled siRNA using Lipofectamine 3000 Transfection Reagent (Invitrogen) in Opti-MEM Reduced Serum Medium (Invitrogen) for 12 h. The transfected cells were then treated with or without Huaier for 48 h. Cell viability was assessed using a CCK-8 kit. Three independent biological replicates were performed for the experiments. The sequences of siRNA are listed in Table S8.

### Virus preparation and viral infection

To prepare lentivirus for knockdown experiments, HEK293T cells were transduced with the pSicoR vector along with the lentivirus packaging vectors PSPAX2 and pMD2G using the calcium phosphate–DNA co-precipitation method. For lentivirus preparation aimed at protein expression, HEK293T cells were transduced with the pCDH-CAG-MCS-IRES-mCherry vector and the same packaging vectors. Medium containing the virus was collected 48 h after transfection. Cells were then infected with the collected viral supernatant in the presence of polybrene (8 µg/ml).

### Overexpression of SLC7A11 and GPX4 in NSCLC cells

For stable overexpression of SLC7A11 and GPX4 in A549, H1975, and H358 cells, RNA isolated from A549 cells was reverse transcribed into cDNA. The human cDNA of SLC7A11 and GPX4 was cloned into the pCDH-CAG-mCherry lentivirus vector. All constructs generated were confirmed by DNA sequencing.

### Lactate dehydrogenase release assay

Lactate dehydrogenase (LDH) leakage was conducted to determine cell injury. The released LDH was detected in the collected culture medium using spectrophotometry with the CytoTox 96 non-Radioactive Cytotoxicity Assay kit (Promega, Madison, USA).

### Measurement of ROS and malondialdehyde (MDA)

Cellular ROS was measured by flow cytometry using HDCF-DA from Sigma Aldrich (D6883). MDA, a major indicator of lipid peroxidation, was measured using a kit (#M496, Dojindo, Kumamoto, Japan) according to the manufacturer’s instructions and normalized based on the protein concentration.

### Cystine uptake assay

Cystine uptake was measured using the Cystine Uptake Assay Kit-WST (#UP05, Dojindo, Kumamoto, Japan) according to the manufacturer’s protocol. This kit enables the measurement of xCT activity through fluorescence, offering a more straightforward alternative to radiolabeled methods. The Cystine Analog (CA) contained in the kit is taken up into cells via xCT, and the xCT activity is measured by detecting the fluorescence of the CA.

### Glutamate measurement

Glutamate concentration was determined using the Glutamate Assay Kit-WST (Dojindo, Kumamoto, Japan) according to the manufacturer’s protocol. Briefly, glutamate standards or samples from the cell culture medium were added to a 96-well microplate, followed by the addition of the working solution to each well. After incubating the microplate at 37 °C for 30 min, the absorbance was measured at 450 nm. The glutamate concentration in each sample was calculated by referencing a standard curve.

### Localization of intracellular Fe^2+^

Cells were seeded in glass-bottom 24-well plates and allowed to grow overnight. After treatment as indicated in the main figure, cells were co-stained with FerroOrange (5 μM) and LysoTracker Green (50 nM) in serum-free medium for 30 min at 37 °C in an incubator. Subsequently, the cells were washed with PBS. Digital images were captured and analyzed for further evaluation.

### Measurement of lipid peroxidation using Liperfluo

Lipid peroxides were analyzed using Liperfluo probe labeling. Cells were plated in 6-well plates at a density of 1.5 × 10^5^ cells/well. After treatment, cells were washed twice with 1×PBS and then stained with 5 μM Liperfluo dye (diluted in Serum-free medium) for 30 min at 37 °C in an incubator. Post-staining, the cells were washed with PBS, and digital images were captured and analyzed for further investigation.

### Urethane induced primary lung cancer models

All animal experiments were performed in accordance with the guidelines of the Ethical Review Committee for Experimental Animal Welfare, Institute of Zoology, Chinese Academy of Sciences (approval number: IOZ-1ACUC-2022-024). Female Balb/c mice (5–6 weeks old) were purchased from SPF Biotechnology co., Ltd (Beijing, China). In the lung cancer therapy group, the Balb/c mice were exposed to urethane (800 mg/kg twice per week for 5 weeks) in 0.9% NaCl via intraperitoneal injection. On day 180, Micro-CT scans were performed on the mice’s lungs. Mice with the same number and size of lung tumors were selected and then daily gavaged with Huaier or vehicle (0.9% NaCl). Subsequent Micro-CT scans were conducted at 180 days, 210 days, and 240 days, with the mice sacrificed around 240 days for the isolation and analysis of lung tissues.

In the lung cancer prevention group, the Balb/c mice were exposed to urethane (800 mg/kg twice per week for 5 weeks) in 0.9% NaCl via intraperitoneal and treated with Huaier (0, 0.5, 1.5, 4.5 g/kg) via oral gavage per day for two to six months. For Micro-CT (PerkinElmer, Waltham, MA) analyses, the survival of the mice was evaluated from the first day of treatment until death or when they became moribund, at which point the mice were sacrificed.

### Tumor xenograft experiments

Immune-deficient BALB/c nude mice (4–5 weeks old) were purchased from SPF Biotechnology Co., Ltd (Beijing, China). These mice were used to establish A549 xenografts by injecting 6 × 10^6^ A549 cells. The mice were exposed to SRS16-86 (2 mg/kg) twice per week and then treated daily with Huaier (4.5 g/kg) by gavage for 24 consecutive days. All animal experiments were performed in accordance with the guidelines of the Ethical Review Committee for Experimental Animal Welfare, Institute of Zoology, Chinese Academy of Sciences (approval number: IOZ-1ACUC-2022-024).

### Long-term toxicity experiments

Female Balb/c mice, aged 8 weeks, were obtained from SPF Biotechnology Co., Ltd in Beijing, China. They were administered Huaier at a dose of 4.5 g/kg daily via gavage for a duration of three months. Post-treatment, the liver and kidneys were harvested, fixed in 4% paraformaldehyde, and embedded in paraffin for histopathological evaluation. Pathological changes were then examined using an inverted microscope. All animal experiments adhered to the guidelines set by the Ethical Review Committee for Experimental Animal Welfare at the Institute of Zoology, Chinese Academy of Sciences, under approval number IOZ-1ACUC-2022-024.

### Statistical analysis

The data are the means of at least three independent experiments and are presented as mean ± standard deviation (SD), except specific clarification. The significance of the results was determined using GraphPad Prism software (San Diego, CA, USA). For comparisons between two groups, Student’s *t*-test was used. For comparisons among more than two groups, one-way analysis of variance (ANOVA) was used. For comparison of survival curves, the log-rank test was performed. *p < 0.05, **p < 0.01, ***p < 0.001, and ****p < 0.0001 were considered significant.

## Supplementary information

Supplementary Figure legends

Supplementary Figures 1

Supplementary Figures 2

Supplementary Figures 3

Supplementary Figures 4

Supplementary Figures 5

Supplementary Tables (1-8)

Full and uncropped western blots

## Data Availability

All data supporting the findings of this study are provided within the article and its Supplementary Information file.
