# Supplementary material for: Huaier suppresses lung cancer by simultaneously and independently inhibiting the antioxidant pathway SLC7A11/GPX4 while enhancing ferritinophagy
Source: Cell Death Discov. 2025 Jul 7;11:309. doi: 10.1038/s41420-025-02598-3 (PMC12234692; doi:10.1038/s41420-025-02598-3)
Supplement: Supplementary file 1 — Supplementary Figure legends [file 41420_2025_2598_MOESM1_ESM.docx]

**Figure S1. Huaier inhibits the progression of lung cancer and exhibits no significant toxic effects on mice in long-term toxicity experiments.**

**(a)** Representative images of lung surface, with yellow triangles marking primary lung tumors. n=3. **(b)** A schematic diagram illustrates the establishment of the long-term toxicity experiment in mice. n=6. **(c)** Changes of mouse weight. Histopathological examination of the kidney **(d)** and liver **(e)** tissues by H&E staining. Scale bar: 50 µm.

**Figure S2. Huaier Induces Ferroptosis in NSCLC Cells.**

**(a)** Huaier-induced cell death in NSCLC cell lines (A549, H1975, and H358) treated with Huaier (2.5, 5, or 10 mg/ml) for 48 h. Representative images are shown. Scale bar: 100 μm. **(b)** Huaier inhibites cell proliferation in a dose-dependent manner. A549, H1975, and H358 cells were treated with Huaier at concentrations of 0, 1, 3, 5, 7.5, 10, and 15 mg/ml for 48 h, and the inhibition ratio was measured using the CCK-8 assay. **(c)** Huaier enhances Fe^2+^ accumulation in NSCLC cells, as quantified by the fluorescence intensity of FerroOrange. Data are shown as mean ± SD, n =3, ***p* < 0.01, ****p* < 0.001. **(d)** Huaier increases lipid peroxidation in NSCLC cells. Representative FACS data show Liperfluo labeling of A549, H1975, and H358 cells treated with Huaier at 5 or 10 mg/ml for 48 h. **(e)** Lipid peroxidation was detected in A549, H1975, and H358 cells treated with Huaier for 48 hours using Liperfluo staining. Scale bar: 100 μm. **(f)** LDH levels in the cell supernatant were measured in A549, H1975, and H358 cells treated with Huaier at 3, 5, or 10 mg/ml for 48 hours. Data are presented as mean ± SD, n=3, **p* < 0.05, ***p* < 0.01, ****p* < 0.001.  **(g)** A549, H1975, and H358 cells were treated with Huaier at 5 or 10 mg/ml for 48 hours and then stained with DCFH-DA for flow cytometry analysis. Data are presented as mean ± SD, n=3, ***p* < 0.01, ****p* < 0.001. **(h)** Intracellular MDA contents were measured in A549, H1975, and H358 cells treated with Huaier at 5 mg/ml in the absence or presence of Ferrostatin-1 (Fer-1) at 2 μM for 48 h. Data are shown as mean ± SD, n =3, ***p* < 0.01, ****p* < 0.001.

**Figure S3. Huaier Induces Ferroptosis of NSCLC Cells via SLC7A11/GPX4 Axle**

**(a)** Western blot analysis of GPX4 (left) and SLC7A11 (right) expression in A549, H1975, and H358 cells following treatment with Huaier at concentrations for 48 h. β-actin served as a loading control. Data are shown as mean ± SD, n =3, ****p* < 0.001, *****p* < 0.0001. **(b)** Confocal imaging of GPX4 (red) in A549, H1975, and H358 cells treated with Huaier (5 mg/ml) for 48 h, with nuclei stained using DAPI stain (blue). Scale bar: 20 μm. **(c)** Huaier suppresses cystine uptake in NSCLC cells, as measured in A549, H1975, and H358 cells treated with Huaier (5 mg/ml) for 48 h. Data are shown as mean ± SD, n =3, ***p* < 0.01. **(d)** Glutamate levels were measured in A549, H1975, and H358 cells treated with huaier (5 mg/ml) for 48 h. Data are shown as mean ± SD, n =3, **p* < 0.05, ***p* < 0.01, ****p* < 0.001. **(e-g)** A549 cells were transfected with 100 nM specific GPX4 siRNA (*siGPX4*) and then treated with Huaier (5 mg/ml) for 48 h. **(e, f)** GPX4 expression at mRNA and protein levels were measured. β-actin served as a loading control. Data are shown as mean ± SD, n =3, **p* < 0.05, ***p* < 0.01. **(g)** Cell viability was assessed in A549, H1975, and H358 cells following Huaier (5 mg/ml) treatment with or without *siGPX4* for 48 h.Data are shown as mean ± SD, n =3, **p* < 0.05, ***p* < 0.01, ****p* < 0.001. **(h)** GPX4 expression was examined by western blot after treatment of A549, H1975, and H358 cells with RSL3 (5 μM) for 48 h. β-actin served as a loading control. Data are shown as mean ± SD, n =3, **p* < 0.05, ***p* < 0.01. **(i)** Cell viability was assessed in A549, H1975, and H358 cells following Huaier (5 mg/ml) treatment with or without RSL3 (5 μM) for 48 h. Data are shown as mean ± SD, n =3, **p* < 0.05, ****p* < 0.001. **(j)** SLC7A11 was overexpressed in A549, H1975, and H358 cells, and its expression was analyzed by western blot. β-actin served as a loading control. Data are shown as mean ± SD, n =3, **p* < 0.05, ***p* < 0.01. **(k)** A549, H1975, and H358 cells were stably transfected with GPX4 plasmid, and GPX4 expression was analyzed by western blot. β-actin served as a loading control. Data are shown as mean ± SD, n =3, **p* < 0.05, ***p* < 0.01. **(l)** In A549 and H358 cells, stably transfected with the SLC7A11 plasmid and treated with Huaier at 5 mg/ml for 48 h, Western blot analysis was performed to evaluate SLC7A11 and GPX4 protein levels. β-actin served as a loading control. Data are shown as mean ± SD, n =3, **p* < 0.05, ****p* < 0.001, *****p* < 0.0001. **(m)** These cells were then treated with Huaier (5 mg/ml) for 48 h, and lipid peroxidation was detected using Liperfluo staining. Scale bar: 100 μm. **(n)** A549, H1975, and H358 cells were stably transfected with a GPX4 plasmid and treated with Huaier (0, 1, 3, 5, 7.5, 10 mg/ml) for 48 h, with the inhibition ratio assessed using a CCK-8 assay.

**Figure S4. Huaier Induces Ferritinophagy in NSCLC Cells**

**(a)** Western blot analysis of NCOA4 and FTH1 expression in A549 cells treated with Huaier at concentrations of 0, 5, 10 mg/ml for 48 h. β-actin served as a loading control. Data are shown as mean ± SD, n =3, **p* < 0.05, *****p* < 0.0001. **(b)** Western blot analysis of NCOA4 and FTH1 expression in A549 cells treated with 5 mg/ml Huaier for 0, 12, 24, and 48 h. β-actin served as a loading control. Data are shown as mean ± SD, n =3, ***p* < 0.01. ****p* < 0.001, *****p* < 0.0001. **(c, d)** Western blot analysis of NCOA4 and FTH1 expression in H1975 and H358 cells treated with Huaier (5 mg/ml) for 48 h. β-actin served as a loading control. Data are shown as mean ± SD, n =3, ***p* < 0.01, ****p* < 0.001. **(e)** Huaier does not affect the mRNA expression levels of FTH1 and FTL in NSCLC cells. A549, H1975, and H358 cells were treated with Huaier (5 mg/ml) for 0, 6, 12, 24,48 h. **(f, g)** Confocal imaging of FTH1 (red), LC3 (green) and LAMP1 (green) in A549 cells treated with Huaier (5 mg/ml) for 48 h. LC3 marks autophagosome, while LAMP1 stains lysosomes (green). Colocalized foci are highlighted by white boxes and shown in enlarged images. Scale bar: 10 μm. **(h)** Confocal imaging of NCOA4 (red) and LAMP1 (green) in A549 cells treated with Huaier at concentration of 5 mg/ml for 48 h. LAMP1 stains lysosomes, with colocalized foci indicated by white boxes and shown in enlarged images. Scale bar: 20 μm. **(i)** Confocal imaging of FerroOrange (red) and LysoTracker Green (green) in A549 cells treated with Huaier (5 mg/ml) for 48 h. Colocalized foci are indicated by white boxes and shown in enlarged images. Scale bar: 5 μm. **(j)** Quantification of the fluorescence intensity of FerroOrange colocalized with LysoTracker Green per cell, with approximately 100 cells measured per condition. Data are shown as mean ± SD, ****p* < 0.001. **(k, l)** Western blot analysis of NCOA4 and FTH1 expression levels in A549 cells following treatment with Huaier (0, 5, 10 mg/ml) with or without 3-MA (5 mM) or BafA1 (100 nM) for 48 h. β-actin served as a loading control. Data are shown as mean ± SD, n =3, ns (no significance), **p* < 0.05, ***p* < 0.01, ****p* < 0.001, *****p* < 0.0001.

**Figure S5.** **Huaier Simultaneously and Independently Downregulates the SLC7A11/GPX4 Axis and Upregulates Ferritinophagy**

**(a)** A549, H1975, and H358 cells were transfected with 100 nM of either negative control siRNA (siNC) or specific *NCOA4* siRNA (*siNCOA4*). The GPX4 mRNA levels were measured, with data shown as mean ± SD, n =3, ***p* < 0.01, ****p* < 0.001. **(b)** A549 cells were transfected with 100 nM of specific NCOA4 siRNA (*siNCOA4*), and subsequently treated with 5 mg/ml Huaier for 48 h. Western blot analysis of NCOA4 and FTH1 protein levels. β-actin served as a loading control. Data are shown as mean ± SD, n =3, ***p* < 0.01, ****p* < 0.001. **(c)** These cells were transfected with 100 nM siNC or *siNCOA4* for 24 h before being exposed to Huaier (3 or 5 mg/ml) for 48 h. Lipid peroxidation was assessed using Liperfluo staining. Scale bar: 100 μm. **(d)** Representative FACS data show Liperfluo labeling of A549 cells treated with Huaier (5 mg/ml) with or without 3-MA (5 mM) for 48 h. **(e)** Overexpression of SLC7A11 does not influence NCOA4-mediated FTH1 degradation. A549 cells were stably transfected with SLC7A11 plasmids, and the expression of LC3B-I/II, NCOA4, and FTH1 was analyzed via western blot. β-actin served as a loading control. Data are shown as mean ± SD, n =3, ns (no significance), **p* < 0.05, ***p* < 0.01, *****p* < 0.0001. **(f)** *NCOA4* knockdown does not affect expression of SLC7A11 and GPX4. A549 cells were transfected with 100 nM siNC or *siNCOA4* for 24 h before exposure to Huaier (5 mg/ml) for 48 h, followed by Western blot analysis of SLC7A11 and GPX4 expression levels. β-actin served as a loading control. Data are shown as mean ± SD, n =3, ***p* < 0.01. **(g, h)** Western blot analysis of SLC7A11 and GPX4 expression levels in A549 cells treated with Huaier (3 or 5 mg/ml) with or without BafA1 (100 nM) for 48 h. β-actin served as a loading control. Data are shown as mean ± SD, n =3, ***p* < 0.01, *****p* < 0.0001. **(i)** Lung tissues from the mice treated as Figure 1a were isolated at day 240 and analyzed. The expression levels of GPX4, SLC7A11, FTH1 and NOCA4 in tumor tissues were determined by western blot. n = 3 mice per group.β-actin served as a loading control. Data are shown as mean ± SD, n =3, **p* < 0.05, ***p* < 0.01.
