# Supplementary material for: Huaier suppresses lung cancer by simultaneously and independently inhibiting the antioxidant pathway SLC7A11/GPX4 while enhancing ferritinophagy
Source: Cell Death Discov. 2025 Jul 7;11:309. doi: 10.1038/s41420-025-02598-3 (PMC12234692; doi:10.1038/s41420-025-02598-3)
Supplement: Supplementary file 2 — Supplementary Figures 1 [file 41420_2025_2598_MOESM2_ESM.pptx]

## Slide 1
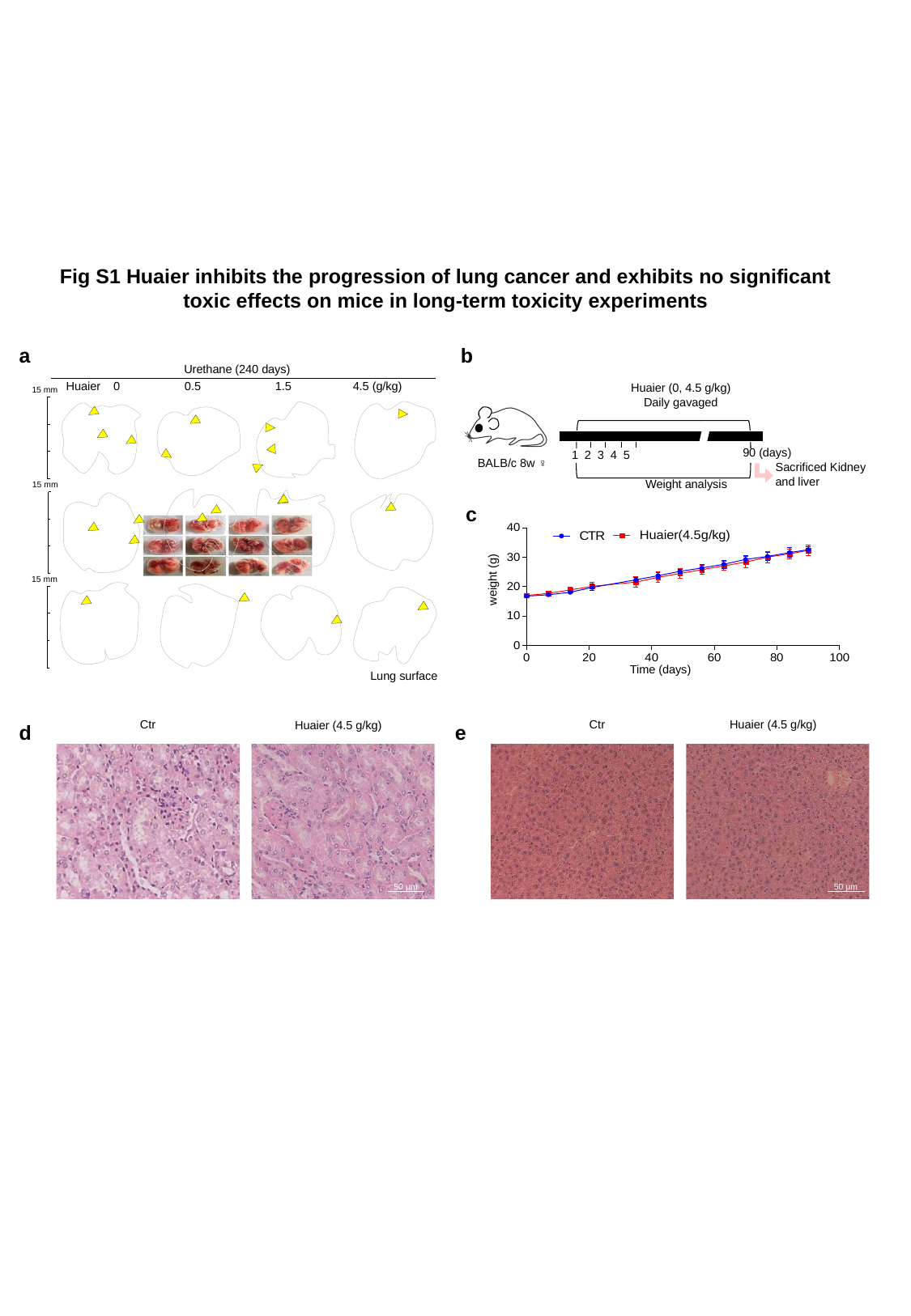

Fig S1 Huaier inhibits the progression of lung cancer and exhibits no significant toxic effects on mice in long-term toxicity experiments
b
Huaier (0, 4.5 g/kg)
Daily gavaged
90 (days)
Weight analysis
Time (days)
1 2 3 4 5
BALB/c 8w ♀
Sacrificed Kidney and liver
a
Urethane (240 days)
 Huaier 0 0.5 1.5 4.5 (g/kg)
15 mm
15 mm
c
weight (g)
15 mm
Lung surface
Ctr
Ctr
Huaier (4.5 g/kg)
Huaier (4.5 g/kg)
d e
50 μm
50 μm
