# Supplementary material for: Huaier suppresses lung cancer by simultaneously and independently inhibiting the antioxidant pathway SLC7A11/GPX4 while enhancing ferritinophagy
Source: Cell Death Discov. 2025 Jul 7;11:309. doi: 10.1038/s41420-025-02598-3 (PMC12234692; doi:10.1038/s41420-025-02598-3)
Supplement: Supplementary file 3 — Supplementary Figures 2 [file 41420_2025_2598_MOESM3_ESM.pptx]

## Slide 1
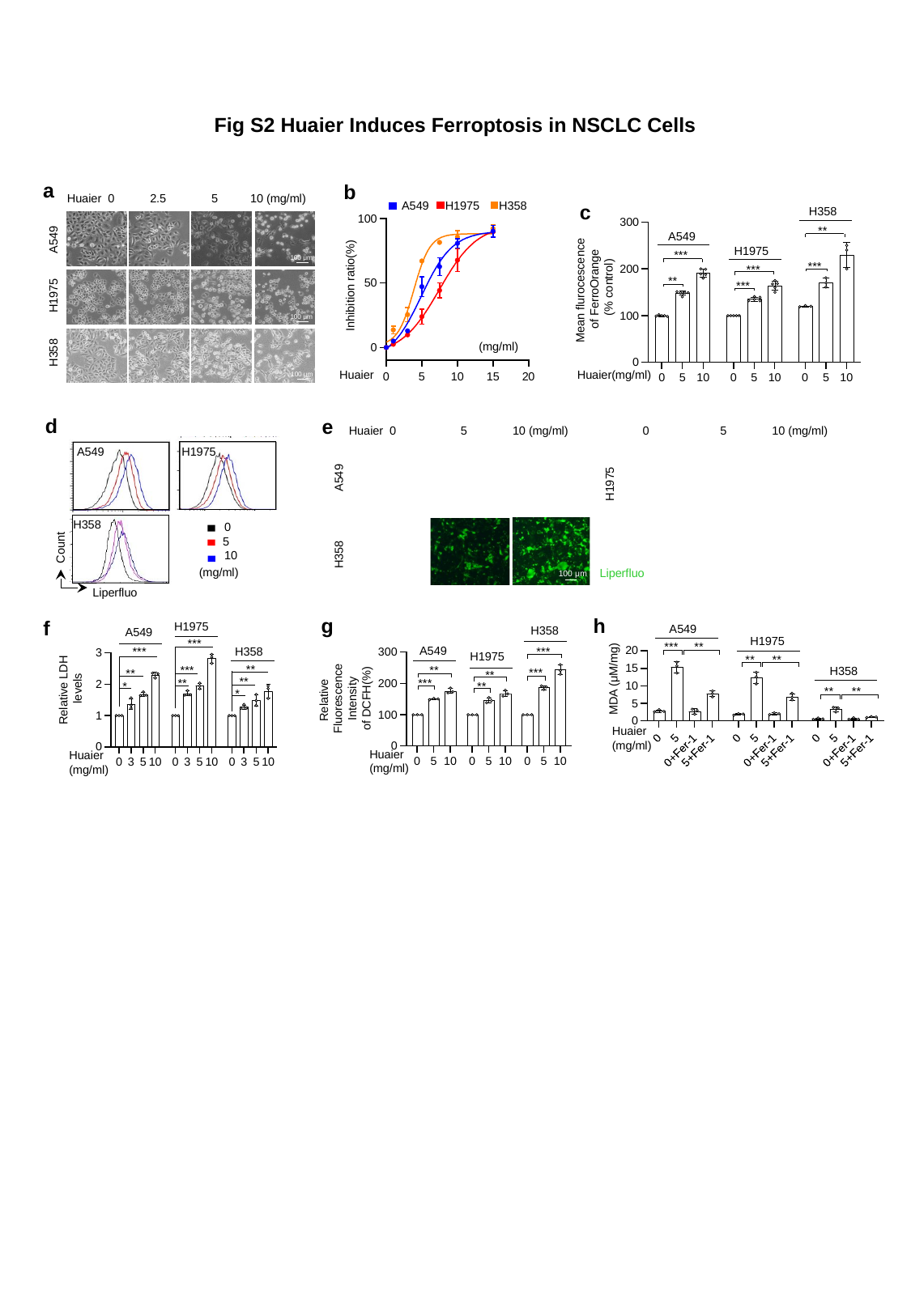

Fig S2 Huaier Induces Ferroptosis in NSCLC Cells
a
b
Huaier 0 2.5 5 10 (mg/ml)
A549
H358 H1975
c
**
***
***
***
***
Mean flurocescence
of FerroOrange
 (% control)
**
Huaier(mg/ml)
A549 H1975 H358
H358
A549
H1975
100 μm
Inhibition ratio(%)
100 μm
(mg/ml)
Huaier
100 μm
d
A549
H1975
H358
0
5
10
(mg/ml)
Count
Liperfluo
e
Huaier 0 5 10 (mg/ml) 0 5 10 (mg/ml)
A549
H1975
H358
Liperfluo
100 μm
100 μm
100 μm
g
h
f
H1975
A549
H358
A549
H1975
***
 **
**
***
***
A549
***
***
***
H358
H1975
**
 **
**
***
H358
*
**
**
**
**
**
Relative
Fluorescence
 Intensity
of DCFH(%)
MDA (μM/mg)
Relative LDH
 levels
*
**
 **
Huaier
(mg/ml)
Huaier
(mg/ml)
Huaier
(mg/ml)
