# Supplementary material for: Huaier suppresses lung cancer by simultaneously and independently inhibiting the antioxidant pathway SLC7A11/GPX4 while enhancing ferritinophagy
Source: Cell Death Discov. 2025 Jul 7;11:309. doi: 10.1038/s41420-025-02598-3 (PMC12234692; doi:10.1038/s41420-025-02598-3)
Supplement: Supplementary file 4 — Supplementary Figures 3 [file 41420_2025_2598_MOESM4_ESM.pptx]

## Slide 1
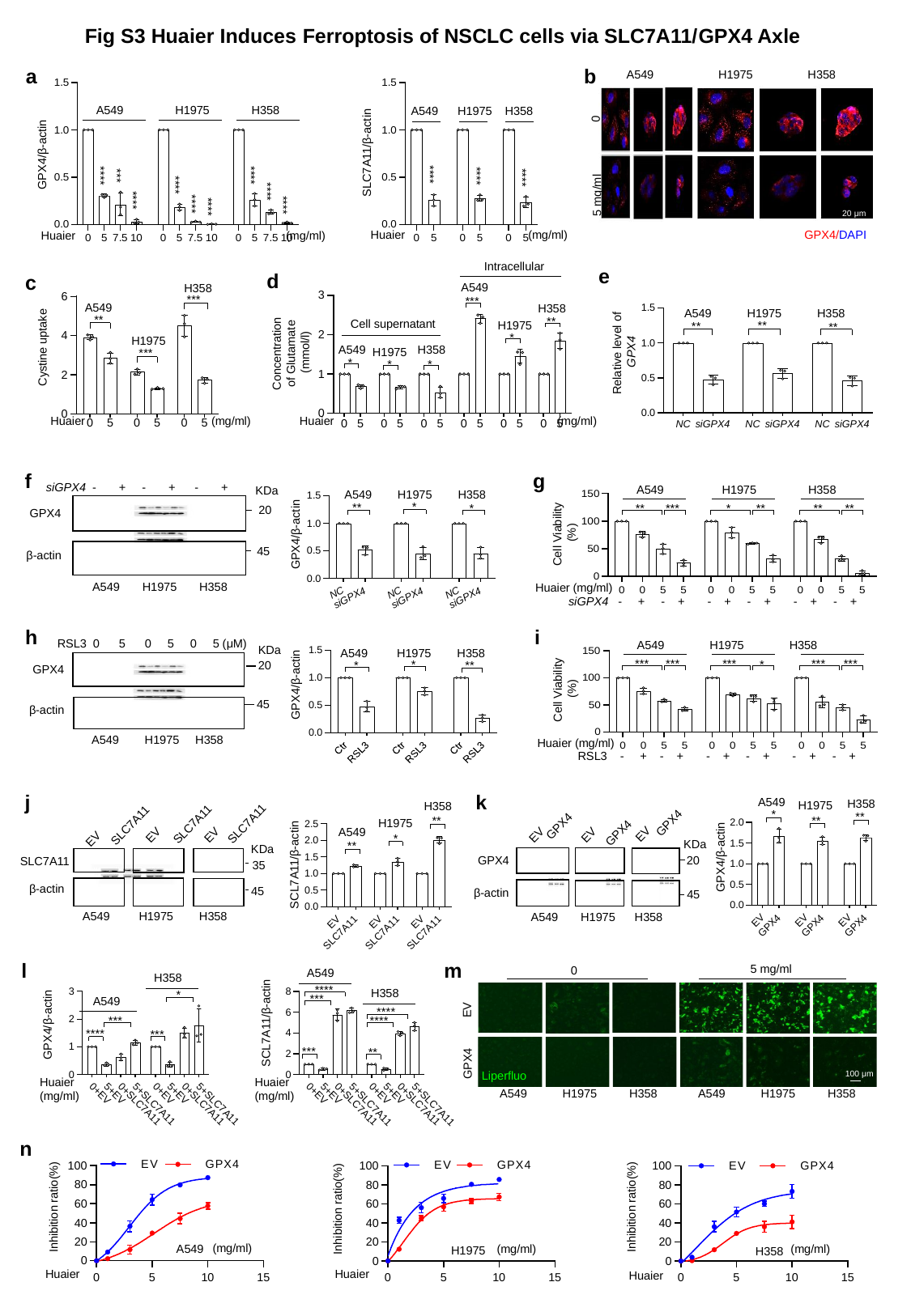

GPX4/DAPI
A549 H1975 H358
5 mg/ml
0
20 μm
Fig S3 Huaier Induces Ferroptosis of NSCLC cells via SLC7A11/GPX4 Axle
a
b
A549 H1975 H358
A549 H1975 H358
SLC7A11/β-actin
GPX4/β-actin
****
****
****
****
****
***
****
****
****
****
****
****
Huaier (mg/ml)
Huaier (mg/ml)
Intracellular
e
d
c
A549
H358
***
***
A549
H358
A549 H1975 H358
**
**
Cell supernatant
H1975
**
**
**
*
Concentration
of Glutamate
(mmol/l)
H1975
Relative level of
GPX4
Cystine uptake
A549
H358
H1975
***
*
*
*
Huaier (mg/ml)
Huaier (mg/ml)
f
g
siGPX4 - + - + - +
GPX4
β-actin
A549 H1975 H358
KDa
20
45
A549 H1975 H358
A549 H1975 H358
*
**
*
***
**
*
**
**
**
Cell Viability
(%)
GPX4/β-actin
Huaier (mg/ml)
siGPX4 - + - + - + - + - + - +
h i
RSL3 0 5 0 5 0 5 (μM)
GPX4
β-actin
 A549 H1975 H358
KDa
20
45
A549 H1975 H358
A549 H1975 H358
***
***
***
***
***
*
*
*
**
Cell Viability
(%)
GPX4/β-actin
Huaier (mg/ml)
 RSL3 - + - + - + - + - + - +
SLC7A11
EV
SLC7A11
β-actin
SLC7A11
SLC7A11
EV
EV
KDa
35
45
A549 H1975 H358
j
k
A549
H358
 GPX4
 GPX4
EV
EV
EV
 GPX4
KDa
20
45
 GPX4
β-actin
A549 H1975 H358
H1975
H358
*
**
**
**
H1975
A549
*
**
GPX4/β-actin
SCL7A11/β-actin
l
m
5 mg/ml
0
EV
 GPX4
A549 H1975 H358
Liperfluo
A549 H1975 H358
A549
H358
****
H358
*
***
A549
****
***
****
SCL7A11/β-actin
GPX4/β-actin
****
***
***
**
100 μm
Huaier
(mg/ml)
Huaier
(mg/ml)
Inhibition ratio(%)
(mg/ml)
H358
Inhibition ratio(%)
Inhibition ratio(%)
(mg/ml)
(mg/ml)
A549
H1975
Huaier
Huaier
Huaier
n
