# Supplementary material for: Huaier suppresses lung cancer by simultaneously and independently inhibiting the antioxidant pathway SLC7A11/GPX4 while enhancing ferritinophagy
Source: Cell Death Discov. 2025 Jul 7;11:309. doi: 10.1038/s41420-025-02598-3 (PMC12234692; doi:10.1038/s41420-025-02598-3)
Supplement: Supplementary file 5 — Supplementary Figures 4 [file 41420_2025_2598_MOESM5_ESM.pptx]

## Slide 1
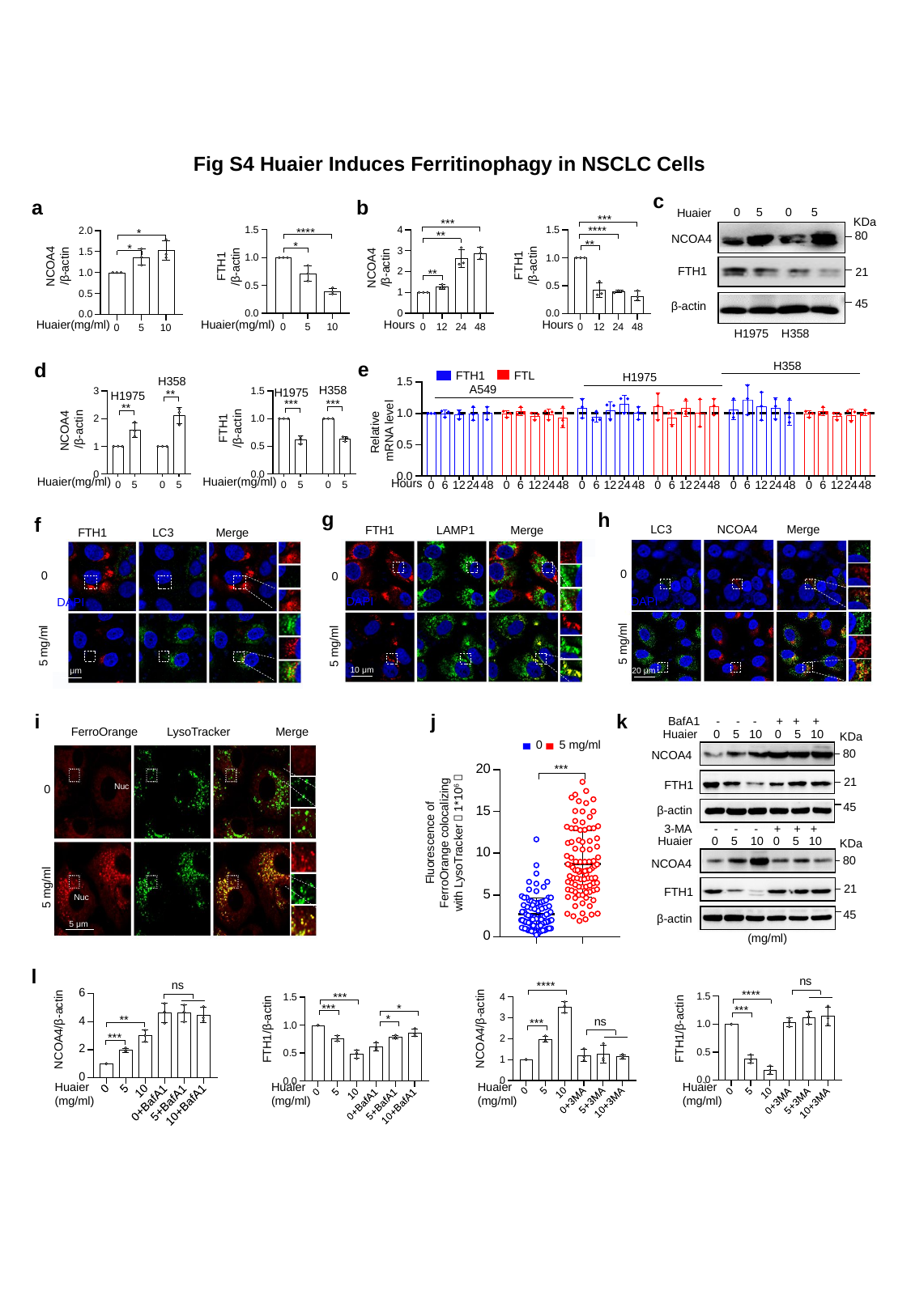

Fig S4 Huaier Induces Ferritinophagy in NSCLC Cells
c
a
b
0 5 0 5
NCOA4
FTH1
β-actin
Huaier
H1975 H358
KDa
80
21
45
***
***
****
****
*
**
**
*
*
FTH1
/β-actin
NCOA4
/β-actin
FTH1
/β-actin
NCOA4
/β-actin
**
Huaier(mg/ml)
Hours
Hours
Huaier(mg/ml)
e
d
 H358
 H1975
 A549
Relative
 mRNA level
Hours
FTL
FTH1
H358
H358
H1975
**
H1975
***
***
**
FTH1
/β-actin
NCOA4
/β-actin
Huaier(mg/ml)
Huaier(mg/ml)
g
FTH1 LAMP1 Merge
0
DAPI
5 mg/ml
10 μm
h
LC3 NCOA4 Merge
0
5 mg/ml
DAPI
20 μm
f
FTH1 LC3 Merge
0
DAPI
5 mg/ml
10 μm
i
j
k
BafA1 - - - + + +
Huaier 0 5 10 0 5 10
KDa
80
21
45
NCOA4
FTH1
β-actin
 3-MA - - - + + +
Huaier 0 5 10 0 5 10
NCOA4
FTH1
β-actin
KDa
80
21
45
5 mg/ml
0
***
Fluorescence of
 FerroOrange colocalizing
with LysoTracker（1*106）
FerroOrange LysoTracker Merge
Nuc
0
5 mg/ml
Nuc
5 μm
(mg/ml)
l
ns
ns
****
****
***
***
*
***
*
**
ns
***
NCOA4/β-actin
FTH1/β-actin
NCOA4/β-actin
FTH1/β-actin
***
Huaier
(mg/ml)
Huaier
(mg/ml)
Huaier
(mg/ml)
Huaier
(mg/ml)
