# Supplementary material for: Huaier suppresses lung cancer by simultaneously and independently inhibiting the antioxidant pathway SLC7A11/GPX4 while enhancing ferritinophagy
Source: Cell Death Discov. 2025 Jul 7;11:309. doi: 10.1038/s41420-025-02598-3 (PMC12234692; doi:10.1038/s41420-025-02598-3)
Supplement: Supplementary file 6 — Supplementary Figures 5 [file 41420_2025_2598_MOESM6_ESM.pptx]

## Slide 1
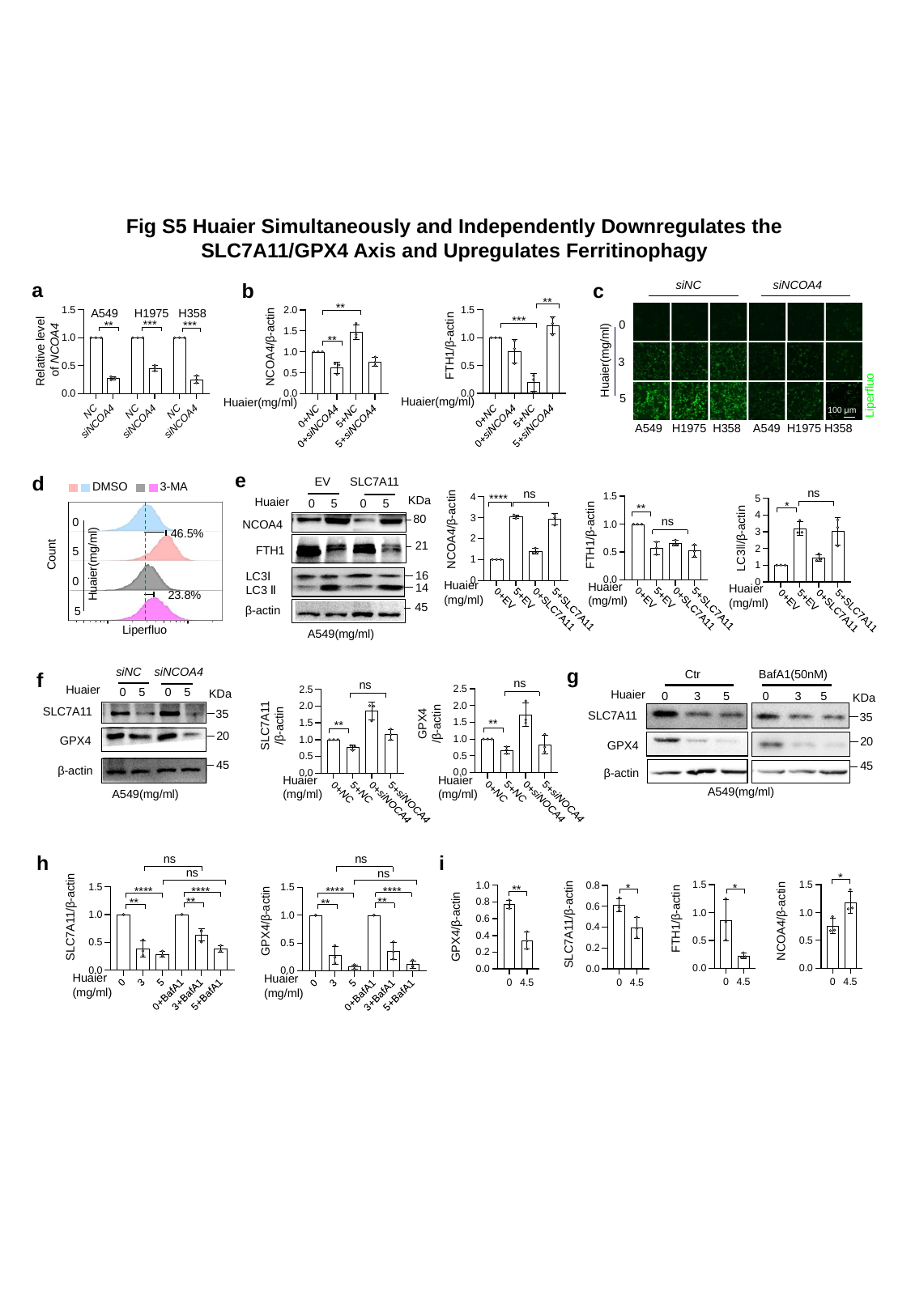

Fig S5 Huaier Simultaneously and Independently Downregulates the SLC7A11/GPX4 Axis and Upregulates Ferritinophagy
a
siNC
siNCOA4
0
3
Liperfluo
5
A549 H1975 H358 A549 H1975 H358
 b c
**
**
A549 H1975 H358
***
***
***
**
**
Relative level
of NCOA4
FTH1/β-actin
NCOA4/β-actin
Huaier(mg/ml)
Huaier(mg/ml)
Huaier(mg/ml)
100 μm
e
d
DMSO 3-MA
0
46.5%
Count
5
0
23.8%
5
Liperfluo
 EV SLC7A11
KDa
Huaier
0 5 0 5
80
NCOA4
21
FTH1
16
LC3Ⅰ
LC3 Ⅱ
14
45
β-actin
A549(mg/ml)
ns
ns
****
*
**
ns
NCOA4/β-actin
FTH1/β-actin
LC3Ⅱ/β-actin
Huaier(mg/ml)
Huaier
(mg/ml)
Huaier
(mg/ml)
Huaier
(mg/ml)
siNC siNCOA4
Huaier
 0 5 0 5
KDa
35
20
45
SLC7A11
GPX4
β-actin
A549(mg/ml)
g
Ctr BafA1(50nM)
Huaier
 0 3 5
0 3 5
KDa
35
20
45
SLC7A11
GPX4
β-actin
A549(mg/ml)
f
ns
ns
GPX4
/β-actin
SLC7A11
/β-actin
**
**
Huaier
(mg/ml)
Huaier
(mg/ml)
h
i
ns
ns
ns
ns
*
*
*
**
****
****
****
****
**
**
**
**
SLC7A11/β-actin
FTH1/β-actin
GPX4/β-actin
NCOA4/β-actin
SLC7A11/β-actin
GPX4/β-actin
Huaier
(mg/ml)
Huaier
(mg/ml)
