# Supplementary material for: Huaier suppresses lung cancer by simultaneously and independently inhibiting the antioxidant pathway SLC7A11/GPX4 while enhancing ferritinophagy
Source: Cell Death Discov. 2025 Jul 7;11:309. doi: 10.1038/s41420-025-02598-3 (PMC12234692; doi:10.1038/s41420-025-02598-3)
Supplement: Supplementary file 7 — Supplementary Tables (1-8) [file 41420_2025_2598_MOESM7_ESM.docx]

Table S1. Details of 6 Shared KEGG Pathways Identified by Proteomics and Metabolomics

| **Pathway Name** | **Protein** | **Metabolite** |
| --- | --- | --- |
| Biosynthesis of amino acids | PGK1; ALDOC;  PGK2; PKLR; ASL | S-Adenosylhomocysteine; N-Acetylglutamic acid; L-Valine; L-Tyrosine: L-Phenylalanine; L-Norleucine; L-Glutamine; L-Glutamic acid; L-Cysteine; L-Cystathionine; L-Asparagine; Glycine; Citric acid |
| Carbon  metabolism | PGK1; GPI; ALDOC; PGK2; HK2; ACAT2; OGDHL; ACSS2; PKLR; SDHD | Succinic acid; L-Glutamic acid; L-Cysteine; Glycine; Citric acid; Acetylphosphate |
| Glyoxylate and dicarboxylate metabolism | ACAT2; ACSS2; AFMID | Succinic acid; L-Glutamine;  L-Glutamic acid; Glycine; Citric acid |
| Ferroptosis | HMOX1; SLC7A11; NCOA4; GPX4; MAP1LC3A | L-Glutamic acid, L-Cysteine, Glutathion, Mevalonic acid |
| Tryptophan metabolism | CYP1B1; ACAT2; GCDH; CYP1A1; AFMID; AOX1 | Tryptamine; L-Kynurenine; Indole |
| Metabolic pathways | PGK1; GPI; P4HA1; ALDOC; HMOX1; PGK2; LSS; FADS2; FDPS; ALDH1A3; PTGS2; HK2; ACAT2; PIK3C3; OGDHL; CERS2; ACSS2; ADA; CA9; DCK; MVD; SIRT5; GUCY2C; HMGCR; AOX1; AFMID; LG10; B3GNT2; SDHD; PLCH1; BST1; NANP; PIKFYVE; PEDS1; PANK3; NDUFB1; UGCG; PCYT2; RDH10; PLPP3; VKORC1L1; FADS1; MSMO1; SMPD2; AMY1B; INPP4A; ADSS1; ASL; CYP1A1; MTHFD2L; PKLR; GPX4; CEPT1; CDA; GCDH | Tryptamine; Toluene; Threonic acid; Succinic acid; S-Adenosylhomocysteine; Riboflavin; Pyroglutamic acid; Putrescine; Pseudouridine; Phenylacetaldehyde; Phenol; N-Acetylputrescine; N-Acetylglutamic acid; N-Acetyl-L-aspartic acid; L-Valine; L-Tyrosine; L-Phenylalanine; L-Kynurenine; L-Histidine; L-Glutamine; L-Glutamic acid; L-Cysteine; L-Cystathionine; L-Asparagine; Inosine; Indole; Guanosine; Guanine; Glycine; Glycerol 3-phosphate; Ergothioneine; Cysteinylglycine; Dipropyl disulfide; D-Proline; D-Glucuronic acid; Cyclic GMP; Citric acid; Adenosine monophosphate; Adenine; Acetylphosphate; Acetyl adenylate; 5-Amino-6-ribitylamino uracil; 4-Hydroxycinnamic acid; 4-Hydroxybenzaldehyde; 4-Guanidinobutanoic acid; 3-Methylxanthine; 2-Phenylacetamide; 2-Hydroxycinnamic acid; 1-Methylnicotinamide; |

Table S2. Details of Proteins Identified in the 6 KEGG Signaling Pathways with Expression Changes

| **Proteome Huaier /control** | | | |
| --- | --- | --- | --- |
| **KEGG signaling pathway** | **Protein name** | **Fold change** | **P value** |
| Biosynthesis of amino acids | PGK1 | 1.53 | 1.62E-02 |
|  | ALDOC | 3.83 | 8.11E-03 |
|  | PGK2 | 2.35 | 5.61E-04 |
|  | PKLR | 1.60 | 2.84E-02 |
|  | ASL | 1.61 | 3.53E-02 |
| Carbon metabolism | PGK1 | 1.53 | 1.62E-02 |
|  | SDHD | 0.54 | 2.06E-03 |
|  | PKLR | 1.60 | 2.84E-02 |
|  | OGDHL | 0.37 | 1.12E-03 |
|  | ACSS2 | 1.53 | 2.05E-03 |
|  | ACAT2 | 1.66 | 1.10E-03 |
|  | PGK2 | 2.35 | 5.61E-04 |
|  | HK2 | 2.42 | 4.79E-05 |
|  | ALDOC | 3.83 | 8.11E-03 |
|  | GPI | 1.54 | 1.86E-02 |
| Glyoxylate and dicarboxylate metabolism | ACAT2 | 1.66 | 1.10E-03 |
|  | ACSS2 | 1.53 | 2.05E-03 |
|  | AFMID | 2.27 | 1.54E-03 |
| Ferroptosis | HMOX1 | 1.55 | 2.09E-03 |
|  | SLC7A11 | 0.61 | 1.19E-03 |
|  | GPX4 | 0.63 | 5.54E-04 |
|  | NCOA4 | 2.53 | 6.43E-04 |
|  | MAP1LC3A | 1.51 | 2.95E-04 |
| Tryptophan metabolism | CYP1B1 | 2.82 | 3.98E-06 |
|  | ACAT2 | 1.66 | 1.10E-03 |
|  | GCDH | 0.65 | 3.48E-03 |
|  | CYP1A1 | 4.00 | 3.25E-04 |
|  | AFMID | 2.27 | 1.54E-03 |
|  | AOX1 | 12.81 | 3.78E-02 |
| Metabolic pathways | PGK1 | 1.53 | 1.62E-02 |
|  | GPI | 1.54 | 1.86E-02 |
|  | P4HA1 | 1.66 | 2.29E-03 |
|  | ALDOC | 3.83 | 8.11E-03 |
|  | HMOX1 | 1.55 | 2.09E-03 |
|  | PGK2 | 2.35 | 5.61E-04 |
|  | LSS | 1.58 | 1.43E-03 |
|  | FADS2 | 0.43 | 4.17E-03 |
|  | FDPS | 1.58 | 7.00E-03 |

Table S2. Details of Proteins Identified in the 6 KEGG Signaling Pathways with Expression Changes（Continued）

| **Proteome Huaier /control** | | | |
| --- | --- | --- | --- |
| **KEGG signaling pathway** | **Protein name** | **Fold change** | **P value** |
|  | ALDH1A3 | 1.84 | 1.67E-03 |
|  | PTGS2 | 0.49 | 5.59E-04 |
|  | HK2 | 2.42 | 4.79E-05 |
|  | ACAT2 | 1.66 | 1.10E-03 |
| Metabolic pathways | PIK3C3 | 1.52 | 1.31E-02 |
|  | OGDHL | 0.37 | 1.12E-03 |
|  | CERS2 | 0.55 | 1.17E-03 |
|  | ACSS2 | 1.53 | 2.05E-03 |
|  | ADA | 0.87 | 8.50E-02 |
|  | CA9 | 3.01 | 1.69E-04 |
|  | DCK | 1.50 | 1.51E-03 |
|  | MVD | 1.51 | 4.57E-03 |
|  | SIRT5 | 1.55 | 1.91E-04 |
|  | GUCY2C | 1.58 | 1.64E-02 |
|  | HMGCR | 3.47 | 3.74E-04 |
|  | AOX1 | 12.81 | 3.78E-02 |
|  | AFMID | 2.27 | 1.54E-03 |
|  | ALG10 | 0.55 | 1.47E-02 |
|  | B3GNT2 | 2.00 | 5.65E-04 |
|  | SDHD | 0.54 | 2.06E-03 |
|  | PLCH1 | 0.59 | 3.97E-03 |
|  | BST1 | 0.60 | 2.93E-03 |
|  | NANP | 0.62 | 4.48E-02 |
|  | PIKFYVE | 1.57 | 2.16E-03 |
|  | PEDS1 | 1.64 | 1.09E-03 |
|  | PANK3 | 1.86 | 5.98E-03 |
|  | NDUFB1 | 0.94 | 3.53E-01 |
|  | UGCG | 2.00 | 1.62E-04 |
|  | PCYT2 | 1.93 | 4.78E-03 |
|  | RDH10 | 1.87 | 2.08E-03 |
|  | PLPP3 | 0.61 | 5.34E-03 |
|  | VKORC1L1 | 0.64 | 1.60E-02 |
|  | FADS1 | 0.54 | 2.01E-03 |
|  | MSMO1 | 1.70 | 6.65E-03 |
|  | SMPD2 | 2.51 | 4.62E-03 |
|  | AMY1B | 1.61 | 5.72E-03 |
|  | INPP4A | 1.57 | 1.21E-02 |
|  | ADSS1 | 1.93 | 3.32E-03 |
| Table S2. Details of Proteins Identified in the 6 KEGG Signaling Pathways with Expression Changes（Continued） | | | |
| **Proteome Huaier /control** | | | |
| **KEGG signaling pathway** | **Protein name** | **Fold change** | **P value** |
|  | ASL | 1.61 | 3.53E-02 |
|  | CYP1A1 | 4.00 | 3.25E-04 |
|  | MTHFD2L | 0.62 | 1.37E-02 |
|  | PKLR | 1.60 | 2.84E-02 |
| Metabolic pathways | GPX4 | 0.63 | 5.54E-04 |
|  | CEPT1 | 0.59 | 3.00E-03 |
|  | CDA | 2.11 | 3.41E-02 |
|  | GCDH | 0.65 | 3.48E-03 |

Table S3. Details of Metabolites Identified in the 6 KEGG Signaling Pathways with Changes

| **Metabolome Huaier /control** | | | |
| --- | --- | --- | --- |
| **KEGG signaling pathway** | **Metabolite name** | **Fold change** | **P value** |
| Biosynthesis of amino acids | S-Adenosylhomocysteine | 0.46 | 1.34E-02 |
|  | N-Acetylglutamic acid | 0.25 | 2.15E-03 |
|  | L-Valine | 0.80 | 1.72E-02 |
|  | L-Tyrosine | 0.71 | 4.79E-03 |
|  | L-Phenylalanine | 0.81 | 8.30E-03 |
|  | L-Norleucine | 0.93 | 6.67E-01 |
|  | L-Glutamine | 0.71 | 2.27E-03 |
|  | L-Glutamic acid | 0.62 | 1.76E-02 |
|  | L-Cysteine | 0.43 | 1.25E-03 |
|  | L-Cystathionine | 4.40 | 1.70E-03 |
|  | L-Asparagine | 0.87 | 2.44E-02 |
|  | Glycine | 0.62 | 1.11E-03 |
|  | Citric acid | 13.30 | 2.06E-02 |
| Carbon metabolism | Succinic acid | 0.84 | 4.35E-02 |
|  | L-Glutamic acid | 0.62 | 1.76E-02 |
|  | L-Cysteine | 0.43 | 1.25E-03 |
|  | Glycine | 0.62 | 1.11E-03 |
|  | Citric acid | 13.30 | 2.06E-02 |
|  | Acetylphosphate | 1.99 | 1.08E-02 |
| Glyoxylate and dicarboxylate metabolism | Succinic acid | 0.84 | 4.35E-02 |
|  | L-Glutamine | 0.71 | 2.27E-03 |
|  | L-Glutamic acid | 0.62 | 1.76E-02 |
|  | Glycine | 0.62 | 1.11E-03 |
|  | Citric acid | 13.30 | 2.06E-02 |
| Ferroptosis | L-Glutamic acid | 0.62 | 1.76E-02 |
|  | L-Cysteine | 0.43 | 1.25E-03 |
|  | Glutathione | 0.58 | 2.44E-01 |
| Tryptophan metabolism | Tryptamine | 5.54 | 5.89E-04 |
|  | L-Kynurenine | 0.77 | 2.66E-02 |
|  | Indole | 0.71 | 3.02E-02 |
| Metabolic pathways | Uridine diphosphate glucuronic acid | 0.58 | 6.69E-06 |
|  | Uracil | 0.43 | 1.72E-04 |
|  | Tryptamine | 5.54 | 5.89E-04 |
|  | Toluene | 0.83 | 2.31E-02 |

Table S3. Details of Metabolites Identified in the 6 KEGG Signaling Pathways with Changes（Continued）

| **Metabolome Huaier /control** | | | |
| --- | --- | --- | --- |
| **KEGG signaling pathway** | **Metabolite name** | **Fold change** | **P value** |
| Metabolic pathways | Threonic acid | 0.37 | 2.84E-02 |
|  | Succinic acid | 0.84 | 4.35E-02 |
|  | S-Adenosylhomocysteine | 1.33 | 5.73E-01 |
|  | Riboflavin | 0.69 | 7.42E-03 |
|  | Pyroglutamic acid | 0.05 | 9.77E-04 |
|  | Putrescine | 0.63 | 1.61E-02 |
|  | Pseudouridine | 0.46 | 2.06E-03 |
|  | Phenylacetaldehyde | 0.66 | 1.76E-02 |
|  | Phenol | 0.81 | 3.68E-02 |
|  | N-Acetylputrescine | 0.72 | 1.31E-02 |
|  | N-Acetylglutamic acid | 0.25 | 2.15E-03 |
|  | N-Acetyl-L-aspartic acid | 0.47 | 2.05E-03 |
|  | L-Valine | 0.80 | 1.72E-02 |
|  | L-Tyrosine | 0.71 | 4.79E-03 |
|  | L-Phenylalanine | 0.81 | 8.30E-03 |
|  | L-Kynurenine | 0.77 | 2.66E-02 |
|  | L-Histidine | 0.85 | 4.57E-02 |
|  | L-Glutamine | 0.71 | 2.27E-03 |
|  | L-Glutamic acid | 0.62 | 1.76E-02 |
|  | L-Cysteine | 0.43 | 1.25E-03 |
|  | L-Cystathionine | 4.40 | 1.70E-03 |
|  | L-Asparagine | 0.87 | 2.44E-02 |
|  | Inosine | 1.76 | 1.88E-04 |
|  | Indole | 0.71 | 3.02E-02 |
|  | Guanosine | 1.53 | 5.12E-03 |
|  | Guanine | 0.66 | 2.82E-02 |
|  | Glycine | 0.62 | 1.11E-03 |
|  | Glycerol 3-phosphate | 0.57 | 6.35E-03 |
|  | Ergothioneine | 7.31 | 6.68E-05 |
|  | Cysteinylglycine | 0.56 | 6.22E-03 |
|  | Dipropyl disulfide | 0.45 | 4.72E-02 |
|  | D-Proline | 0.61 | 6.98E-03 |
|  | D-Glucuronic acid | 0.09 | 3.85E-04 |
|  | Cyclic GMP | 0.42 | 1.82E-03 |
|  | Citric acid | 13.30 | 2.06E-02 |
|  | Adenosine monophosphate | 0.49 | 3.58E-03 |

Table S3. Details of Metabolites Identified in the 6 KEGG Signaling Pathways with Changes（Continued）

| **Metabolome Huaier /control** | | | |
| --- | --- | --- | --- |
| **KEGG signaling pathway** | **Metabolite name** | **Fold change** | **P value** |
| Metabolic pathways | Adenine | 0.85 | 4.64E-02 |
|  | Acetylphosphate | 1.99 | 1.08E-02 |
|  | Acetyl adenylate | 0.25 | 3.01E-05 |
|  | 5-Amino-6-ribitylamino uracil | 0.65 | 7.29E-03 |
|  | 4-Hydroxycinnamic acid | 0.66 | 2.47E-03 |
|  | 4-Hydroxybenzaldehyde | 0.69 | 1.12E-02 |
|  | 4-Guanidinobutanoic acid | 3228.02 | 5.69E-04 |
|  | 3-Methylxanthine | 3.94 | 8.94E-03 |
|  | 2-Phenylacetamide | 0.69 | 3.09E-03 |
|  | 2-Hydroxycinnamic acid | 0.69 | 8.62E-03 |
|  | 1-Methylnicotinamide | 2.64 | 1.02E-03 |

Table S4. Details of Proteins Identified in the Ferroptosis Pathway with Changes

| **Proteome Huaier /control** | | | | | | | | |
| --- | --- | --- | --- | --- | --- | --- | --- | --- |
| **Protein name** | **Ctronl-1** | **Ctronl-2** | **Ctronl-3** | **Huaier-1** | **Huaier-2** | **Huaier-3** | **Fold change** | **P value** |
| SLC7A11 | 5440.2 | 4910.8 | 4762.1 | 2870.6 | 3294 | 3007.9 | 0.61 | 1.19E-03 |
| GPX4 | 594.9 | 631.8 | 609.4 | 358.8 | 424.9 | 377.3 | 0.63 | 5.54E-04 |
| NCOA4 | 1953.8 | 1855.1 | 1670.9 | 5112.4 | 4152 | 4604.8 | 2.53 | 6.43E-04 |
| FTH1 | 1651.2 | 1854.7 | 1629.5 | 1230.8 | 1241 | 1090 | 0.45 | 3.77E-03 |
| TFRC | 22437.5 | 21627.1 | 20332.9 | 23436.4 | 25266.8 | 20864.7 | 1.08 | 2.91E-01 |
| HMOX1 | 4079 | 4058.3 | 3541.9 | 6534.7 | 5869.8 | 5732.3 | 1.55 | 2.09E-03 |
| ACSL4 | 6940.2 | 6522.8 | 6592.8 | 7914.1 | 7215.6 | 6285.8 | 1.07 | 4.06E-01 |
| GCLC | 7308.7 | 9911.2 | 11700.6 | 8180.8 | 9809.8 | 10044.1 | 0.97 | 8.44E-01 |

Table S5. The List of Reagents Used

| **reagents** | **Cat No.** | **Manufacturer** | **Description** |
| --- | --- | --- | --- |
| Urethane | U2500 | Sigma-Aldrich | mice |
| Carboxy-H2DCFDA | 287810 | Sigma-Aldrich | Cell lines |
| 3-methyladenine(3-MA) | HY-19312 | MCE | Cell lines |
| Bafilomycin A1(BafA1) | HY-100558 | MCE | Cell lines |
| MitoTEMPO | HY-112879 | MCE | Cell lines |
| SRS16-86 | HY-100579 | MCE | mice |
| Deferoxamine (DFO) | HY-B0988 | MCE | Cell lines |
| Ferrostain-1(Fer-1) | HY-100579 | MCE | Cell lines |
| RSL3 | HY-100218A | MCE | Cell lines |
| LysoTracker Green | 40738ES50 | Yeasen | Cell lines |
| Liperfluo | L248 | Dojindo | Cell lines & tissue |
| FerroOrange | F374 | Dojindo | Cell lines |

Table S6. The List of Antibodies Used

| **Antiboby** | **Cat No.** | **Manufacturer** | **Description** |
| --- | --- | --- | --- |
| LC3 | D058-3 | MBL | Immunofluorescence |
| LC3B | 3868 | Cell Signaling Technology | Western blot |
| GPX4 | DF6701 | Affinity | Western blot & Immunofluorescence |
| SLC7A11 | DF12509 | Affinity | Western blot |
| NCOA4 | 66849 | Cell Signaling Technology | Western blot & Immunofluorescence |
| FTH1 | DF4828 | Affinity | Western blot & Immunofluorescence |
| β-actin | A5441 | Sigma-Aldrich | Western blot |
| LAMP1 | 9091 | Cell Signaling Technology | Immunofluorescence |
| HRP-conjugated anti-rabbit IgG | 7074 | Cell Signaling Technology | Western blot |
| HRP conjugated anti-mouse IgG | 7076 | Cell Signaling Technology | Western blot |
| Alexa Fluor 594 anti-rabbit IgG | A-11012 | Invitrogen | Immunofluorescence |
| Alexa Fluor Plus 488 anti-mouse IgG | A32723 | Invitrogen | Immunofluorescence |
| Alexa Fluor Plus 488 anti-rabbit IgG | A32731 | Invitrogen | Immunofluorescence |

Table S7. The List of Primers Used for qRT-PCR or PCR

| **Gene name** | **Forward primer sequence** | | **Reverse primer sequence** | **Description** |
| --- | --- | --- | --- | --- |
| *GPX4* | 5'-GAGGCAAGACCGAAGTAAA  CTAC-3' | 5'-CCGAACTGGTTACACGGG  AA-3' | | qPCR primer |
| *FTH1* | 5'-TCCTACGTTTACCTGTCCA  TGT-3' | 5'-GTTTGTGCAGTTCCAGTA  GTGA-3' | | qPCR primer |
| *ACTIN* | 5'-TGAAGTGTGACGTGGACATC-3' | 5'-GGAGGAGCAATGATCTTGAT-3' | | qPCR primer |
| *FTL* | 5'-TGAAGAGACCGCAAGTGGAA  A-3' | 5'-CAGGATGAATAGCCCCG  ACAA-3' | | qPCR primer |
| *SLC7A11* | 5'-CTAGTCTAGACTAGATGGTCA  GAAAGCCTGTTGTGTCCA-3' | 5'-CGGAATTCGCGTCATAACTT  ATCTTCTTCTGGTACAACT-3' | | PCR primer |
| *GPX4* | 5'-CTAGTCTAGACTAGATGAGCC  TCGGCCGCCTTTGCCG-3' | 5'- CGGAATTCGCGCTAGAAATAGTGGGGCAGGTCCTTCT-3' | | PCR primer |

Table S8. The target sequences of siRNA

|  |
| --- |
|  |

| **Gene name** | **Sequense** | **Description** |
| --- | --- | --- |
| *GPX4* | 5'-GGAGUAACGAAGAGAUCAAAG-3' | Knockdown siRNA oligoes |
| *NCOA4* | 5'-ACTCTTGTTTATCGAAGTATA-3' | Knockdown siRNA oligoes |
| NC | 5'-GUGUAUAAGCUGUACCCAATT-3' | Knockdown siRNA oligoes |
