# Supplementary material for: Huaier suppresses lung cancer by simultaneously and independently inhibiting the antioxidant pathway SLC7A11/GPX4 while enhancing ferritinophagy
Source: Cell Death Discov. 2025 Jul 7;11:309. doi: 10.1038/s41420-025-02598-3 (PMC12234692; doi:10.1038/s41420-025-02598-3)
Supplement: Supplementary file 8 — Full and uncropped western blots [file 41420_2025_2598_MOESM8_ESM.pptx]

## Slide 1
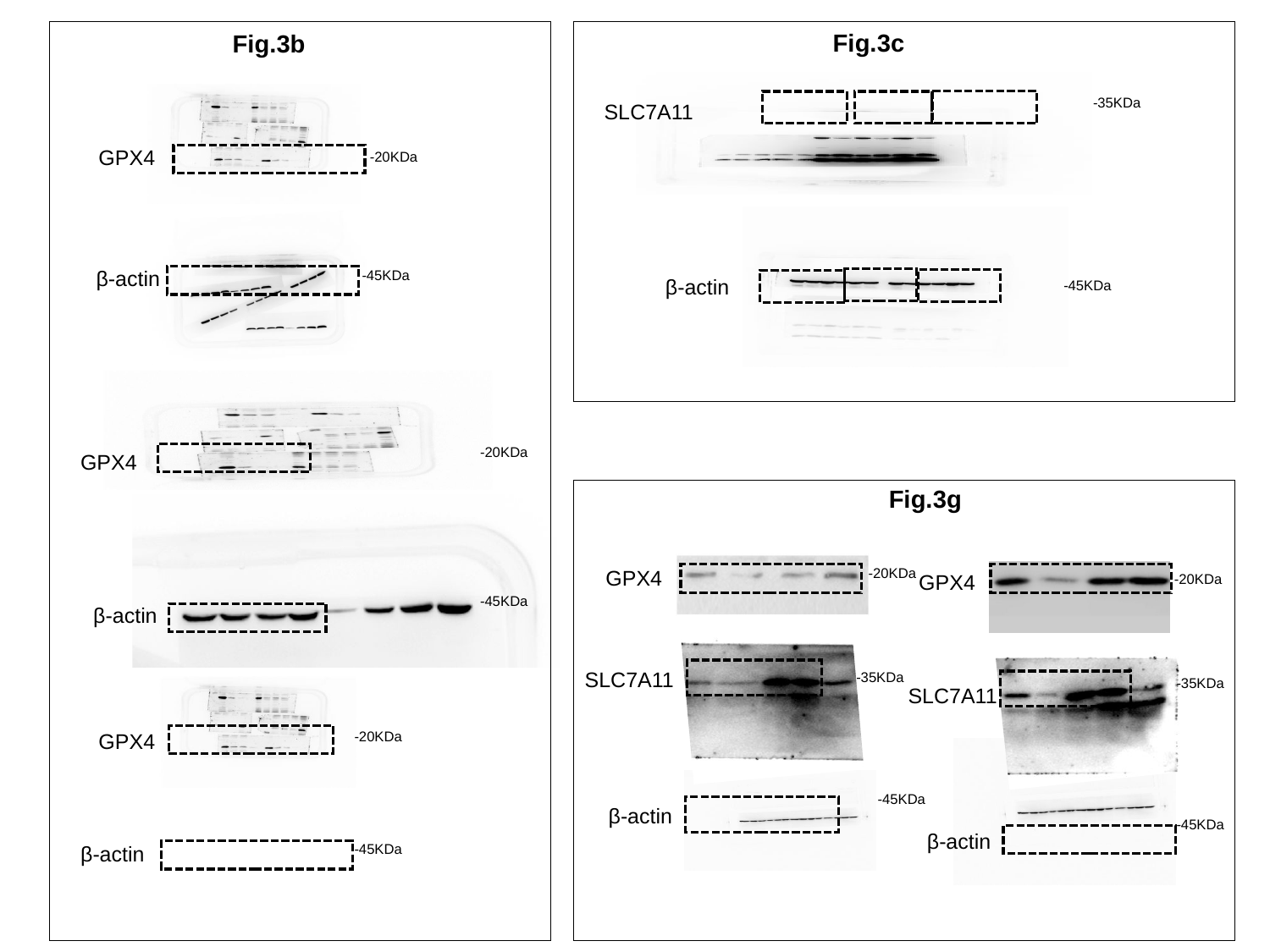

Fig.3c
Fig.3b
GPX4
-20KDa
β-actin
-45KDa
-20KDa
GPX4
-45KDa
β-actin
-20KDa
GPX4
β-actin
-45KDa
-35KDa
SLC7A11
β-actin
-45KDa
Fig.3g
-20KDa
GPX4
GPX4
-20KDa
-35KDa
SLC7A11
-35KDa
SLC7A11
-45KDa
β-actin
-45KDa
β-actin

## Slide 2
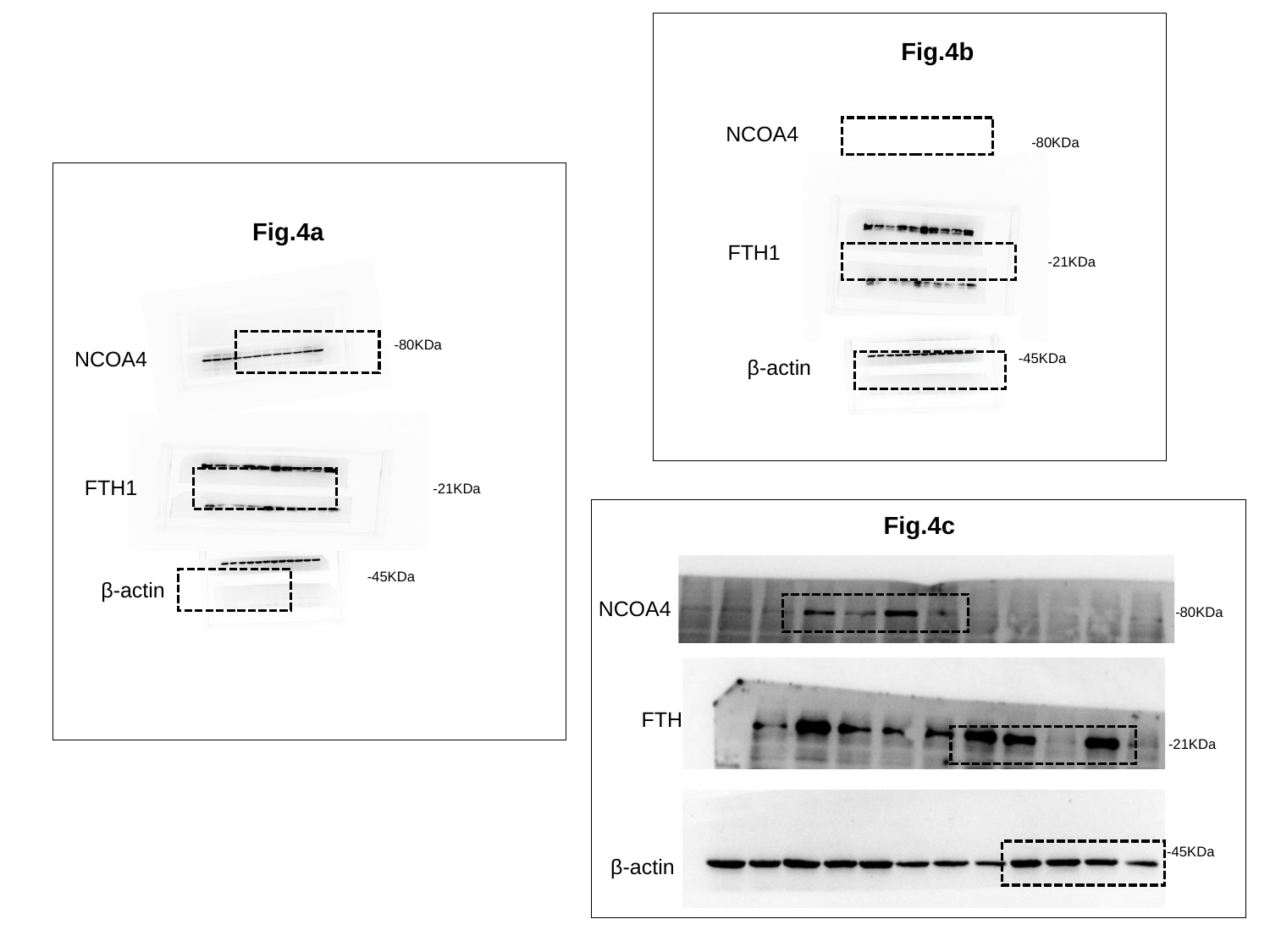

Fig.4b
NCOA4
-80KDa
Fig.4a
FTH1
-21KDa
-80KDa
NCOA4
-45KDa
β-actin
FTH1
-21KDa
Fig.4c
NCOA4
-80KDa
FTH1
-21KDa
-45KDa
β-actin
-45KDa
β-actin

## Slide 3
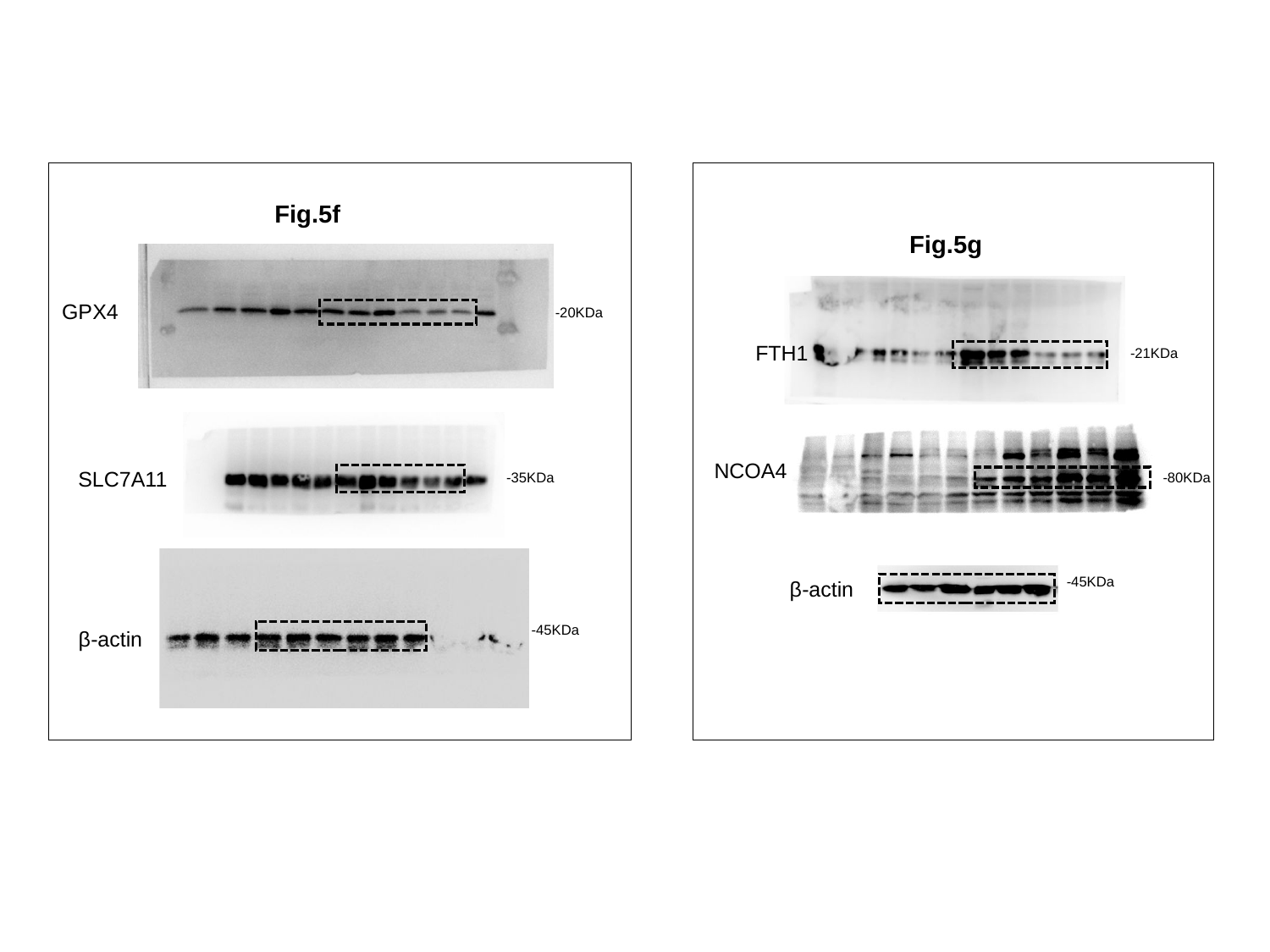

Fig.5f
Fig.5g
GPX4
-20KDa
FTH1
-21KDa
NCOA4
SLC7A11
-80KDa
-35KDa
-45KDa
β-actin
-45KDa
β-actin

## Slide 4
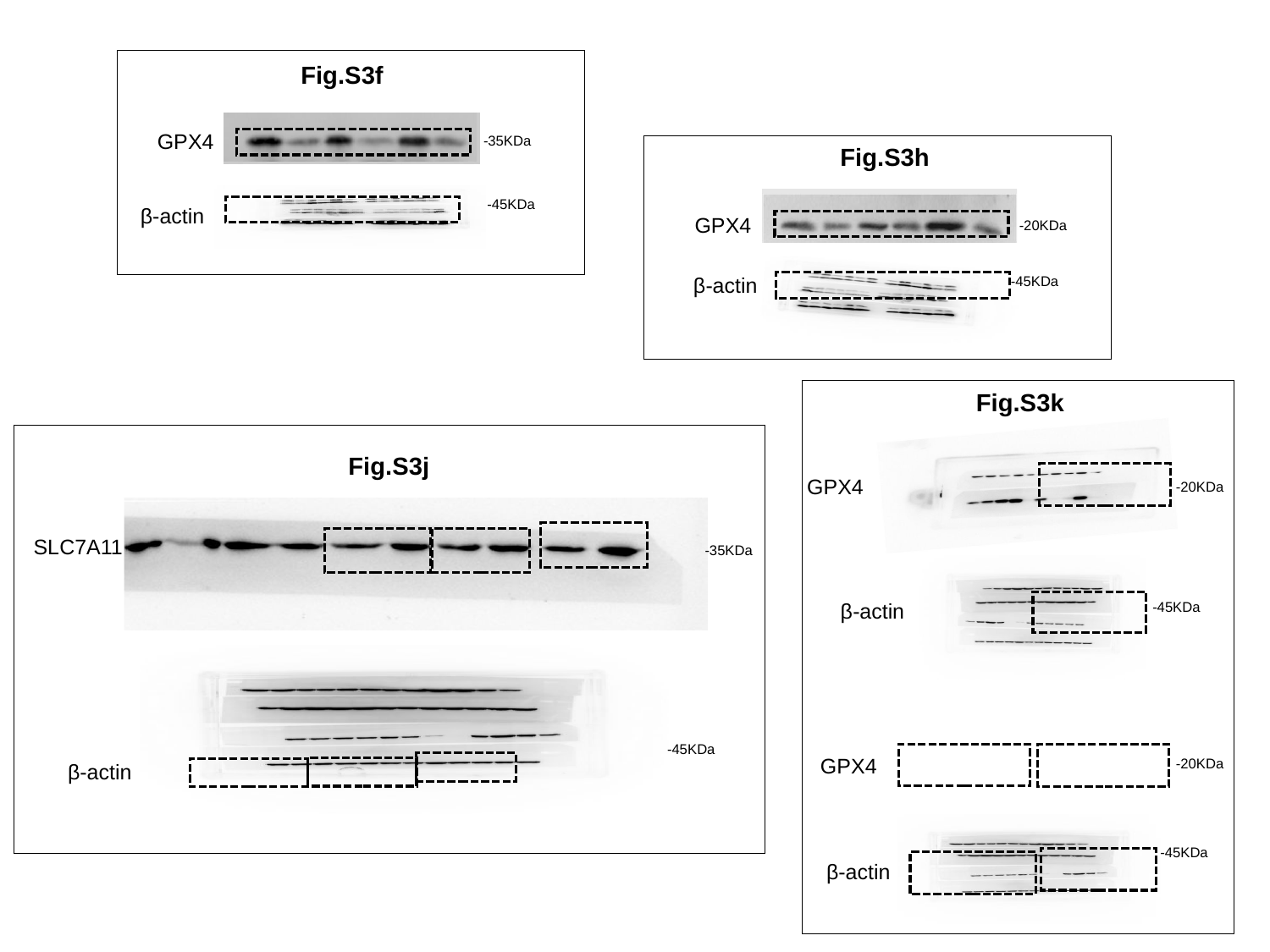

Fig.S3f
GPX4
-35KDa
Fig.S3h
-45KDa
β-actin
GPX4
-20KDa
β-actin
-45KDa
Fig.S3k
Fig.S3j
SLC7A11
-35KDa
-45KDa
β-actin
GPX4
-20KDa
-45KDa
β-actin
GPX4
-20KDa
β-actin
-45KDa

## Slide 5
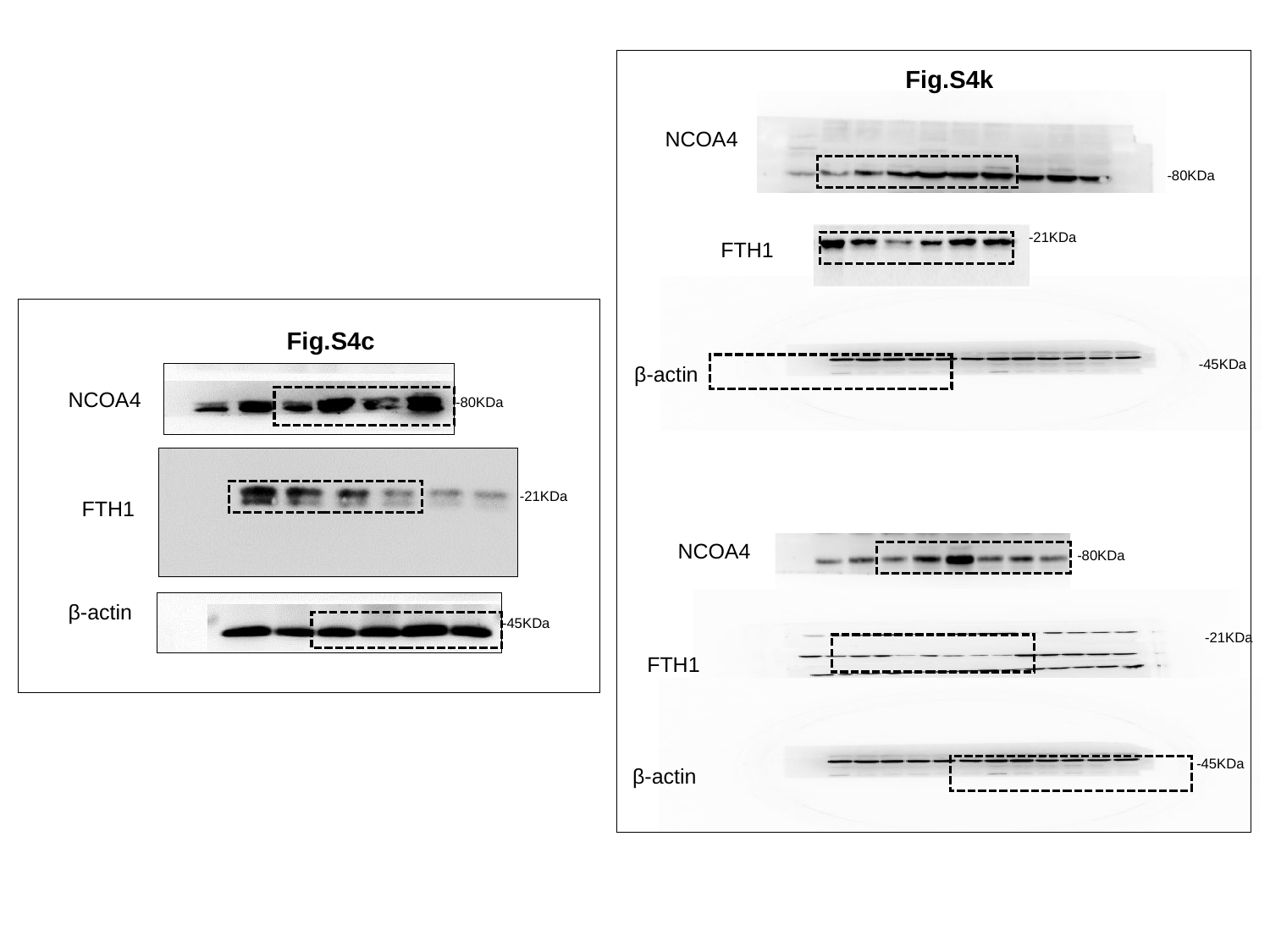

Fig.S4k
NCOA4
-80KDa
-21KDa
FTH1
Fig.S4c
NCOA4
-80KDa
-21KDa
FTH1
β-actin
-45KDa
-45KDa
β-actin
NCOA4
-80KDa
-21KDa
FTH1
-45KDa
β-actin

## Slide 6
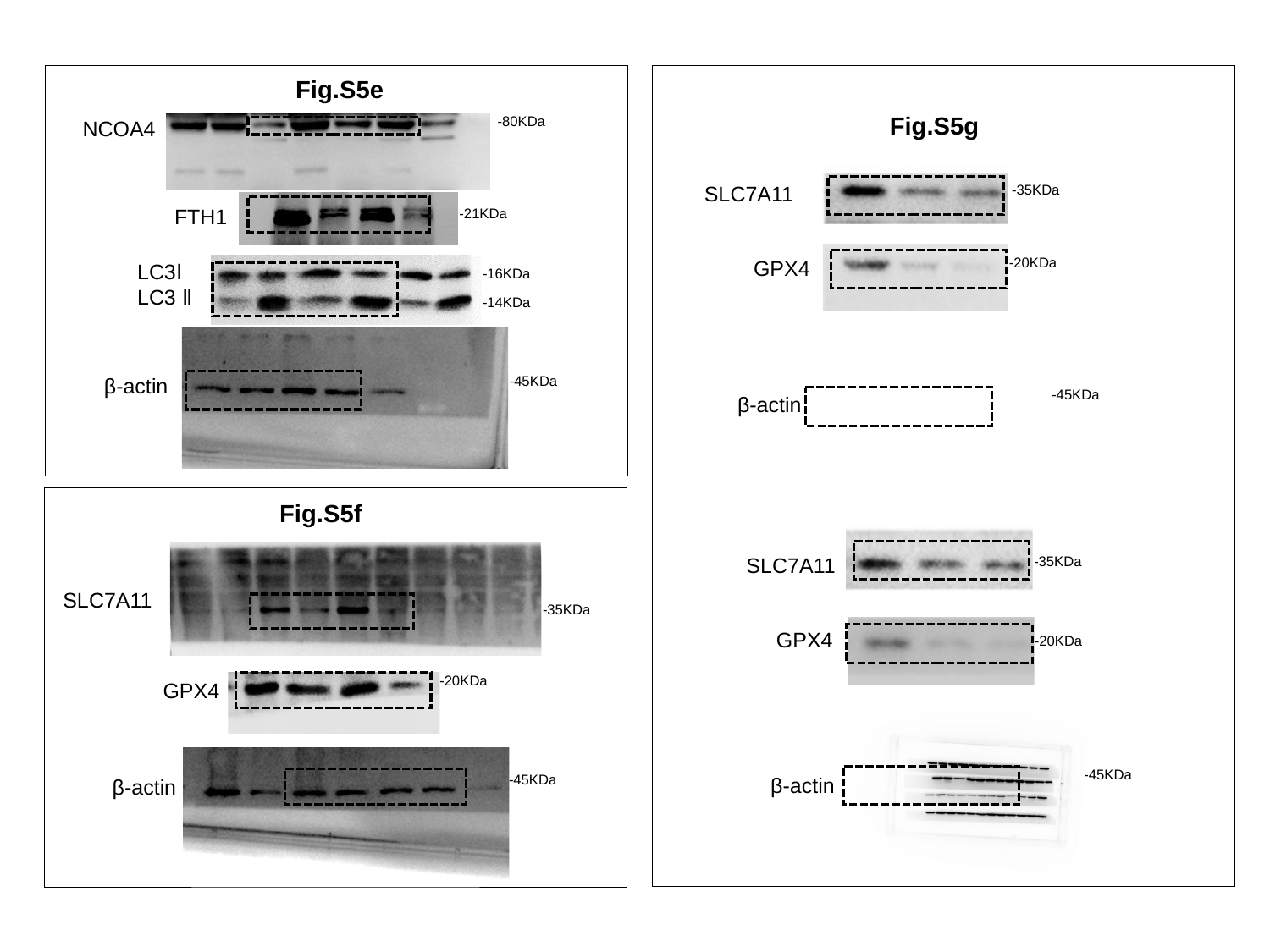

Fig.S5e
-80KDa
NCOA4
FTH1
-21KDa
LC3Ⅰ
LC3 Ⅱ
-16KDa
-14KDa
-45KDa
β-actin
Fig.S5g
SLC7A11
-35KDa
-20KDa
GPX4
-45KDa
β-actin
Fig.S5f
SLC7A11
-35KDa
-20KDa
GPX4
-45KDa
β-actin
SLC7A11
-35KDa
GPX4
-20KDa
-45KDa
β-actin
